# Protocol for a multicentre randomised controlled trial examining the effects of temporarily pausing Bruton tyrosine kinase inhibitor therapy to coincide with SARS-CoV-2 vaccination and its impact on immune responses in patients with chronic lymphocytic leukaemia

**DOI:** 10.1136/bmjopen-2023-077946

**Published:** 2023-09-28

**Authors:** Vicki S Barber, Nicholas Peckham, Lelia Duley, Anne Francis, Abhishek Abhishek, Paul Moss, Jonathan A Cook, Helen M Parry

**Affiliations:** 1 Nuffield Department of Orthopaedics Rheumatology and Musculoskeletal Sciences, Oxford Clinical Trials Research Unit (OCTRU), University of Oxford, Oxford, UK; 2 Nottingham Clinical Trials Unit, University of Nottingham, Nottingham, UK; 3 Academic Rheumatology, University of Nottingham, Nottingham, UK; 4 Institute of Immunology and Immunotherapy, University of Birmingham, Birmingham, UK

**Keywords:** COVID-19, Clinical trials, HAEMATOLOGY

## Abstract

**Introduction:**

People who are immunocompromised have a poor biological response to vaccinations. This study aims to determine in patients with chronic lymphocytic leukaemia (CLL) if a 3-week pause in Bruton tyrosine kinase inhibitor therapy (BTKi) starting 1 week before delivery of SARS-CoV-2 vaccine booster, improves vaccine immune response when compared with continuation of BTKi.

**Methods and analysis:**

An open-label, randomised controlled superiority trial will be conducted in haematology clinics in approximately 10 UK National Health Service (NHS) hospitals. The sample size is 120, randomised 1:1 to intervention and usual care arms. The primary outcome is anti-spike-receptor binding domain (RBD) antibody level at 3 weeks post-SARS-CoV-2 booster vaccination. Secondary outcomes are RBD antibody levels at 12 weeks postbooster vaccination, participant global assessments of disease activity, blood films, full blood count and lactate dehydrogenase levels, impact on quality of life, self-reported adherence with request to temporarily pause or continue BTKi, T cell response against spike protein and relative neutralising antibody titre against SARS-CoV-2 viral variants. Additionally, there will be an investigation of any effects in those given influenza vaccination contemporaneously versus COVID-19 alone.

The primary analysis will be performed on the as randomised groups (‘intention to treat’). The difference between the study arms in anti-spike-RBD antibody level will be estimated using a mixed effects regression model, allowing for repeated measures clustered within participants. The model will be adjusted for randomisation factor (first line or subsequent line of therapy), and prior infection status obtained from prerandomisation antinucleocapsid antibodies as fixed effects.

**Ethics and dissemination:**

This study has been approved by Leeds East Research Ethics Committee and Health Research Authority (REC Reference:22/YH/0226, IRAS ID: 319057). Dissemination will be via peer-review publications, newsletters and conferences. Results will be communicated to participants, the CLL patient and clinical communities and health policy-makers.

**Trial registration number:**

ISRCTN14197181.

**STRENGTHS AND LIMITATIONS OF THIS STUDY**

- It is adequately powered to detect modest differences in anti-spike receptor binding domain (RBD) antibody titres that have been deemed clinically important, alongside what impact there is on the functional quality of responses observed. Therefore, it will provide information about both the benefits and risks of temporarily pausing treatment.
- This is an open-label study so both participants and researchers will be unblinded to group allocation, however, the primary and key secondary outcome measures are laboratory assessed by staff blinded to group allocation.
- The different vaccine types available in the latest vaccination programme may impact the study results—the latest Sanofi vaccination does not contain ancestral SARS-CoV-2 spike protein which is the antigen used in the RBD spike assay on which the primary outcome is based.

## Introduction

Chronic lymphocytic leukaemia (CLL) is the most common adult leukaemia in many countries with 3800 new diagnoses each year in the UK.^1–3^ It is a chronic and currently incurable disease that demonstrates wide clinical heterogeneity.^4 5^ CLL occurs predominantly in older people and is rare below the age of 40 years. An estimated 31 900 people are living with CLL in the UK.^6–8^

People who are immunocompromised have a poor biological response to vaccinations.^9^ CLL is associated with underlying immune suppression. This can lead to considerable risk of infection with associated morbidity and mortality.^10^ Infection remains the cause of death for around 25% of patients with CLL.^11 12^ Most recently, people with CLL were shown to be susceptible to severe COVID-19 and have been prioritised for receiving SARS-CoV-2 vaccines.^13–17^ Unfortunately, vaccine responses in patients with CLL are impaired, particularly in those on current ‘targeted’ drugs (Bruton tyrosine kinase inhibitors (BTKi)) that act to inhibit B cell receptor function. This class of drug is highly effective in suppressing the CLL tumour and is taken continuously in the long term.^18–22^ New approaches to improve immune protection in patients with CLL are needed.^23^ In this study, we seek to investigate if a pragmatic modification of CLL therapy could act to have an impact on vaccine induced immunity in this clinically vulnerable group.

We hypothesise that people taking BTKi specifically Ibrutinib or Acalabrutinib for their CLL at the time of vaccination against COVID-19 (±an influenza vaccination) will have an impaired immune response to the vaccine dose, and therefore, lower production of anti-spike-receptor binding domain (RBD) and neutralising antibodies. We propose that a 3-week pause in BTKi treatment will improve the vaccine-specific immune response, without significant worsening of the control of the underlying CLL, when compared with continuation of treatment as usual. The design and delivery of this study was based around another trial that assessed pausing the immune suppressant drug methotrexate around the time of COVID-19 vaccination in patients with inflammatory disorders.^24^ Pausing of CLL therapy is not uncommon and is recommended perioperatively due to the off-target effect of BTKi therapy on platelet function, which results in an increased bleeding risk.^25 26^ Individuals can then experience a disease flare (which can include enlargement of lymph nodes and/or “B symptoms”, such as fever, sweats and weight loss); most are mild with symptoms such as a fever, rash or mild pain, but some can experience raised lymphocyte counts, enlarged spleens, and pain and changes seen in their blood biochemical profiles.

Currently, no randomised trials have examined the impact of pausing BTKi therapy in patients with CLL for vaccine responsiveness. However, several observational studies have shown poorer responses in those taking BTKi and in addition, improvement in responses off therapy. In the CLL-VR study, following the second dose of vaccine, response rates were almost half in patients who took a BTKi compared with healthy controls.^27^ Greenberger *et al* studied responses to a third dose of COVID-19 vaccination in 25 patients with CLL, 13 of whom were treated with BTKi. Of these, 6/13 had an elevated anti-Spike response but 4 had stopped therapy and one was maintained on a low dose. In comparison, among those who continued therapy, five had a very weak response (<5.2 U/mL) and two had an intermediate response (<250 U/mL).^28^ These data suggest that both the proportion of responders and the strength of response increases in patients not taking BTKi therapy.

Thus, the main aim of this study is to assess whether a 3-week pause in BTKi treatment, 1 week before and 2 weeks after SARS-CoV-2 vaccine booster, improves the vaccine response in people with CLL, compared with continuing BTKi treatment as usual. Key secondary outcomes include any impact on quality of life (QoL) and disease activity.

### Objectives

Primary: Assess the effectiveness of a 3-week pause in BTKi, rather than continuing treatment as usual, on anti-spike-RBD antibody levels at 3 weeks post-SARS-CoV-2 booster vaccination.

Secondary and exploratory: Assess the effectiveness of a 3-week pause in BTKi treatment rather than continuing treatment as usual, on:

- Anti-spike-RBD antibody levels at week 12 after booster vaccination.
- Neutralising antibody titres at baseline and week 3 after booster vaccination against Wuhan D614G and current variant of concern.
- Effect on T cell response to SARS-CoV-2 spike protein at 3 weeks postbooster vaccination.
- Responses to booster vaccination at 3 and 12 weeks after vaccination when influenza vaccination was given at the same time.
- Biochemical disease activity at weeks 3 and 12 postbooster vaccination.
- Disease activity changes and their tolerability as reported by participants during the 12 weeks postbooster vaccination.
- QoL at weeks 3 and 12 after booster vaccination.
- Self-reported adherence to the allocation.

## Methods

### Study design

**Figure 1:**
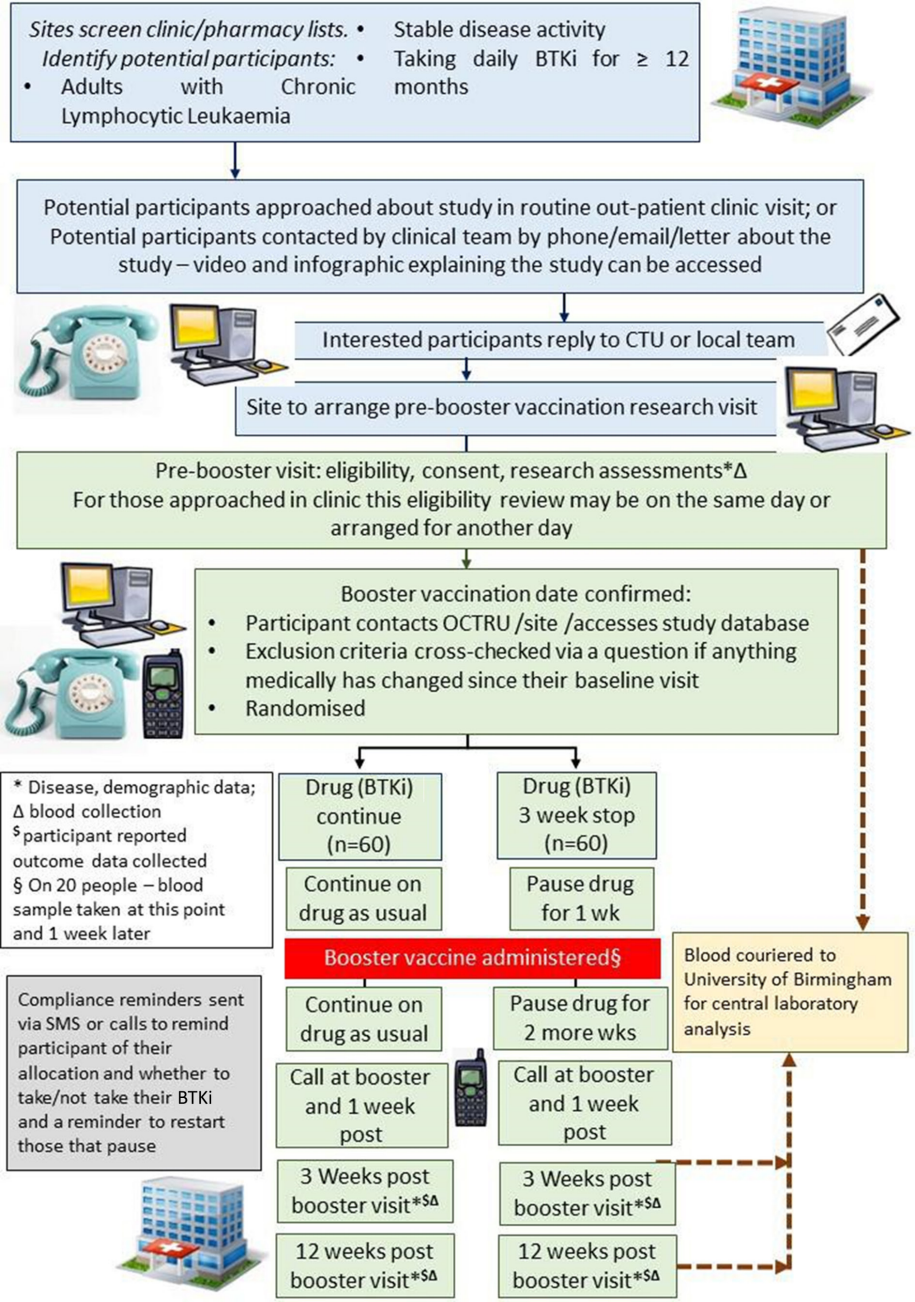
Participant flow in the IMPROVE study. CTU, Clinical Trial Unit; SMS, Short Message Service; BTKi, Bruton tyrosine kinase inhibitor; OCTRU, Oxford Clinical Trials Research Unit.

A two-arm parallel group, multicentre, superiority randomised controlled trial, with 1:1 randomisation. This study will be conducted in approximately 10 NHS hospitals in England and Wales. These will be a mix of district general and university hospitals. Figure 1 provides an overview of the study.

### Recruitment

Participants will be recruited from haematology clinics. Searching of clinic records/hospital/pharmacy databases by the usual care team and identification during routine clinic visits will be the main methods of identification of potentially eligible patients. Social media and promotion of the study by national patient charities, will enable people taking BTKi for their CLL to directly approach their local study teams (or the study centre) to find out more about the study. People identified in the database reviews will be sent a study introductory letter, a participant information leaflet, and a reply slip to return if they are interested in taking part. For those identified directly in clinics, they will be offered the participant information leaflet and the study introductory letter if appropriate.

### Eligibility criteria

#### Inclusion criteria

- Age ≥18 years.
- Confirmed diagnosis of CLL.
- Taking oral BTKi therapy (Acalabrutinib or Ibrutinib) for at least 12 months.
- Has achieved complete remission (including CR with incomplete marrow recovery), partial remission (PR) (including nodular PR or PR with lymphocytosis) or stable disease by the International Workshop on chronic lymphocytic leukaemia response criteria.^29^
- Considered able to pause BTKi therapy for 3 weeks without the risk of substantial increase in disease activity.
- Anticipated to take BTKi over the next 4 months.
- Willing to accept either study arm allocation.
- Able to give informed consent.
- Eligible for a planned booster vaccination for COVID-19.

#### Exclusion criteria

- Insufficient time for applying the intervention prior to the planned COVID-19 booster vaccination.
- Diagnosed with alternative conditions requiring treatment with BTKi.
- Treated with anti-CD20 antibody therapy in the last 18 months or planning to start it.
- Concurrent immune suppressive treatments in the last 3 months specifically: methotrexate, ciclosporin, BCL-2 inhibitors, azathioprine, mycophenolate, prednisolone and biological agents.
- Any contraindications to COVID-19 vaccination.
- Richter’s transformation requiring active therapy.
- Radiotherapy or cancer chemotherapy in last 6 months.
- Active solid organ cancer (people with skin cancer or those cured of solid organ cancer are eligible).
- Receiving or has received in the past 6 months immunoglobulin replacement therapy.
- Receiving or has received in the past 6 months monoclonal antibody therapy against COVID-19 spike protein.

### Randomisation

This will be performed using a centralised validated computer randomisation programme through a secure (encrypted) web-based service, provided by the Oxford Clinical Trials Research Unit (OCTRU). Eligible participants will be randomised when they receive a date for their SARS-CoV-2 booster vaccination. The randomisation system will stratify on whether the participant is on their first line or subsequent line of therapy for CLL to ensure balanced allocation across treatment groups. It will use a 1:1 ratio to allocate to either continuing taking BTKi as usual or to have a 3-week pause (1 week before and 2 weeks after their booster vaccination against COVID-19).

Randomisation will be stratified on the line of CLL therapy being used as it has already been shown that the magnitude of immune response differs depending on the number of lines of therapy.^27^ We have chosen not to stratify on past COVID-19 infection even though it is a strong modifier of serological response to SARS-CoV-2 vaccines as it is difficult to ascertain this reliably from participant self-report. We are also not stratifying on the first vaccine platform received (eg, adenovirus vector or mRNA) as we have previously shown that there is no difference in the magnitude of antibody response by vaccine platform in patients with CLL,^27^ nor on which BTKi therapy the participant is currently taking. However, we will obtain past infection status using anti-nucleocapsid antibodies and use this in the statistical analysis. Due to the nature of the intervention, the participants and the clinical team will not be blind to the allocated arm of the study. However, those analysing the study samples will be blinded to the participants’ allocation. Trial statisticians will not be blinded.

### Treatment arms

The SARS-CoV-2 booster vaccination received while participating in the IMPROVE study will be delivered by the UK’s national vaccination programme.^30^ If another vaccine such as influenza is given at the same time, these data will be recorded.

#### Experimental arm

To suspend BTKi therapy for 1 week before and 2 weeks immediately after receiving the booster vaccination against COVID-19.

#### Control arm

To continue the same dose and regimen of BTKi therapy as usual in the week before and weeks after receiving the booster vaccination against COVID-19.

No concomitant care or interventions are prohibited in the study and the participants’ clinical care team will continue to manage their condition in the usual way after the end of their participation in the study.

Automatic reminders by Short Message Service (SMS) or email will be sent to participants to encourage adherence to their randomised intervention, where they consent to receive these. Participants may be telephoned by the study team if they decline the use of SMS or emails for reminders.

Participants and their usual care team will be able to manage any potential tumour flares including with steroids or any other drug as clinically appropriate. Should a clinical need arise, the participants’ usual care team will be able to advise them to continue or not with their allocation. It is known that tumour flare is fully reversible with the reintroduction of BTKi,^25^ and planned dose suspensions are common and do not appear to compromise long-term outcomes such as progression free or overall survival.^31^

### Outcomes

#### Primary outcome

- SARS-CoV-2 spike RBD-specific antibody titre at 3 weeks postbooster vaccination (PBV).

#### Secondary outcomes

- Levels of anti-spike RBD antibody at 12 weeks PBV.
- Participant assessments of disease activity: global assessment using a Numeric Rating Scale (NRS) with 1-week recall at baseline, 3 and 12 weeks PBV, current disease activity level and change since baseline, 3 and 12 weeks PBV.
- Disease flare-up (tumour flare) and actions taken to deal with them at 3 and 12 weeks PBV.
- Effect on QoL (assessed using European Organisation for Research and Treatment of Cancer -Quality of Life Questionnaire (EORTC-QLQ-CLL17)) at 3 and 12 weeks PBV.
- Adherence with advice to pause or continue BTKi: self-report at 3 weeks PBV.
- COVID-19 neutralising titre at baseline and 3 weeks PBV.
- T cell responses against ancestral Wuhan and the latest variant of concern (B1, B4, B5 and Sanofi) at baseline and 3 weeks PBV.

#### Exploratory outcomes

- Effect on co-administration of influenza vaccination.

#### Safety outcomes

- Serious adverse events (SAEs) that are related to study intervention (recorded from booster vaccination to 12 weeks PBV).

### Data to be collected

Data collection will occur after informed written consent is obtained by a site principal investigator or delegated member of their research team. Participants will also be asked to decide whether to they wish to give an optional consent for storage of any outstanding samples to a Licensed Biobank at the end of the study. Table 1 lists all of the data points and data collected through the study.

**Table 1:** IMPROVE study research assessments at different time points

	Prevaccine (baseline)	Vaccine date known	1 week before vaccination	At vaccination	1 week after vaccination	3 weeks after vaccination	12 weeks after vaccination
Assessments							
Demographics	*						
Height and weight	*						
Current medication	*						
Comorbidities	*						
Past SARS-CoV-2 vaccines	*						
Serum immunoglobins	*						
Disease activity	*					*	*
Quality of life	*					*	*
Randomisation to include a check that no monoclonal antibody has been received since baseline visit		*					
Reminders of allocation to continue or withhold BTKi			*	*	*		
Adherence to intervention						*	
Check on any monoclonal antibody (prophylaxis or treatment for COVID-19)		*				*	*
Safety						*	*
Details of vaccination						*	
Provision of aide memoire card to participant	*						
Thank you note sent after visit	*					*	*
Samples							
Blood sample taken for anti-spike RBD antibody	*					*	*
Blood sample taken for T-cell ELISPOT	*					*	
Blood sample taken for blood film	*					*	*
Blood sample taken for neutralisation assay	*					*	
Blood sample taken for LDH measurement	*					*	*
Blood sample taken for full blood count measurement	*					*	*
Blood sample taken to be banked for mechanistic work as per section 11				†	†	(No extra blood volume required from that already being taken)*	(No extra blood volume required from that already being taken)*

*Indicates timepoint assessment completed.

†To be taken from 20 participants only.

BTKi, Bruton tyrosine kinase inhibitor; LDH, lactate dehydrogenase; RBD, receptor binding domain.

### Baseline visit

Data on demographic factors (age, sex, ethnicity, height, weight, usual residence (home or residential care)). CLL history (number of lines of therapy, which BTKi is taken and starting dates of therapies), CLL disease activity; bloods taken to measure full blood count (FBC) (haemoglobin, platelet count, lymphocyte count); blood film (presence of prolymphocytes, smear cells or lymphocytosis) and lactate dehydrogenase (LDH) levels; smoking status; self-reported physician diagnosis of comorbidities: diabetes including diet-controlled diabetes, hypertension, ischaemic heart disease, congestive cardiac failure, asthma, chronic obstructive pulmonary disease, high cholesterol, stroke including transient ischaemic attack; current medications; past SARS-CoV-2 vaccines will be collected by the local research team. QoL will be assessed using EORTC-QLQ-CLL17.^32^ Patient global assessment of disease activity will be assessed on a 0–10 NRS for past week using the question:

‘Over the LAST 7 DAYS, in all the ways that your CLL affects you, how would you rate the way you have felt?’ In addition to answering yes or no on whether they have had a temperature greater than 37.5°C, and/or noticed new or enlarged lymph nodes.

### Three weeks PBV (±4 days)

Information about any COVID-19 positive testing, a participant’s global assessment of disease activity will be collected, the latter using the same questions asked at the baseline visit asking for the last 7 days and also in the 3-week period since their booster. QoL will be assessed using EORTC-QLQ-CLL17. These data will be generally collected using a link to the study’s online REDCap database sent in a text message, or by email if participant prefers not to use a mobile phone for this purpose. If a participant prefers not to receive this survey link by email or text, this information will be collected by postal questionnaires or at the study visit.

The local research team will collect data at this visit about a participants COVID-19 vaccination history including the most recent COVID-19 vaccination received, details of any other vaccinations coadministered and adherence to the study intervention. In addition, details of any monoclonal antibody treatment will be collected and bloods taken and blood tests undertaken as at baseline. Details of any SAEs will also be recorded.

### Week 12 PBV (±7 days)

Information about any COVID-19 positive testing, any influenza vaccination since the 3-week study visit, the participant’s global assessment of their disease activity will be collected, the latter using the same questions asked at the baseline visit asking about the last 7 days as at week 3. QoL will be assessed using EORTC-QLQ-CLL17 and data will be collected in the methods used at 3 weeks.

The local research team will collect data at this visit about any changes in the participants BTKi prescription, if any NHS services have been sought since the 3-week study visit, or if participants have had any course(s) of steroids since their last visit and details of any monoclonal antibody treatment will be collected. The same bloods and blood tests will be taken as at 3 weeks. Details of any SAEs will also be recorded.

Participants are free to withdraw from the study at any time and all data and samples collected up to the point of withdrawal will be used. Data will be managed and accessible as per the study’s data management plan.

### Sample collection and transport

Blood (10 mL) will be collected in a serum activator tube at baseline, week 3 and 12 visits, along with four lithium heparin tubes (10 mL). These samples will be transported to a central laboratory (the Clinical Immunology Service) at the University of Birmingham by courier. The central laboratory will centrifuge the samples on the day of arrival, aliquot in cryovials and stored at −80°C. They will then be placed into dry ice storage until the analyses are undertaken. For participants recruited at two of the recruiting centres (Birmingham and London), 20 participants will be sought to give optional additional blood samples, specifically the same as collected at the baseline, weeks 3 and 12 visits, within 48 hours and at 1 week PBV.

A further 4 mL EDTA blood tube and 4 mL serum separating blood tube will also be taken at the baseline, 3-week and 12-week visits and processed in the local hospital laboratory to measure FBC and a blood film, and LDH levels accordingly.

#### Laboratory analyses

1. Anti-spike-RBD (primary endpoint at 3 weeks) and anti-nucleocapsid antibodies: Antibody measurements will be undertaken at the University of Birmingham-Clinical Immunology Service (UoB CIS) using validated commercial assays ROCHE-S and ROCHE-N for anti-spike-RBD and antinucleocapsid antibodies, respectively.^33 34^
  ROCHE S refers to the Roche Elecsys Anti-SARS-CoV-2 S immunoassay for the in vitro quantitative determination of antibodies (including IgG) to the SARS-CoV-2 spike protein RBD. The assay uses a recombinant protein representing the RBD of the S antigen in a double-antigen sandwich assay format, which favours detection of high affinity antibodies against SARS-CoV-2.
  ROCHE N refers to the Roche Elecsys Anti-SARS-CoV-2 assay. It uses a modified double-antigen sandwich immunoassay using recombinant nucleocapsid protein (N), which is geared towards the detection of late, mature, high affinity antibodies independent of the subclass. It is a total SARS-CoV-2 antibody assay (IgA, IgM and IgG) detecting predominantly, but not exclusively, IgG.
2. Neutralising antibody titres: Neutralisation assays using pseudotyped virus containing spike protein form Wuhan Hu-1 SARS-CoV-2 and a range of viral variants will be performed at the University of Glasgow on aliquots of the serum collected at baseline and at 3 weeks. All experiments will be conducted in duplicate and absorbance readings will be standardised against positive and negative controls and averaged. Neutralisation curves will be plotted, with the percentage neutralisation modelled as a logistic function of the serum dilution factor (log10). A non-linear regression (curve fit) method will be used to determine the dilution fold that neutralised 50% (IC50) of samples.
3. T cell ELIspot analysis: T cell ELIspot analysis will be performed at the University of Birmingham to detect IFN gamma cytokine release by T cells after extraction of PBMCs from the whole blood received in the lithium heparin tube at baseline and at 3 weeks. All experiments will be conducted in duplicate and absorbance readings will be standardised against positive and negative controls and averaged.
4. Serum immunoglobulins: Serum immunoglobulins will be analysed by the UoB CIS. Quantification of IgG, IgA and IgM will be evaluated using COBAS 6000 (Roche).
5. Anti-influenza antibodies: Serum anti-influenza antibodies will be analysed by the UoB CIS. Specifically, these will be measured by ELISA for 4 H1N1 antigens to reflect the current vaccine component for those that have received a influenza vaccine.

### Data protection and confidentiality

Personal information about potential and enrolled participants will be collected and processed securely, in compliance with the Data Protection Act (DPA) and General Data Protection Regulation (GDPR).

### Sample size and justification

A total of 120 participants will be randomised. The sample size estimates were based on the anti-spike-RBD response after vaccination dose from a small observational study of patients with CLL who were either taking or not taking BTKi.^27^ This suggested a large suppressive effect of BTKi (effect size of 0.9SD). However, anti-spike RBD response related to booster vaccination in CLL patients suggests a smaller though still large effect is more plausible (circa 0.7 SD). Conservatively, we have based the sample size calculation on an effect size of 0.65SD (detecting at least a 0.97 unit (log10 scale) higher antibody response in the BTKi suspension group with 90% statistical power at two-sided 5% significance level which requires data from 102 participants. This leads to 120 participants after allowing for 15% missing data. This calculation was performed using the Stata V.15.1 ‘power twomeans’ command.

Statistical analysis: Full details will be presented in a separate statistical analysis plan which will be drafted and finalised and aimed to be published prior to the final analysis data lock and will receive review and input from the trial steering committee (TSC) and data monitoring committee. The principal analysis will be performed on the as randomised (‘intention-to-treat’) population, analysing participants with available outcome data in their randomised groups, regardless of adherence. The study will be reported in line with Consolidated Standards of Reporting Trials guidelines.^35^

The primary objective of the statistical analysis will be to identify if a temporary 3-week pausing of BTKi around the time of vaccination against SARS-CoV-2 increases the anti-spike-RBD antibody at 3 weeks postvaccine compared with continuing BTKi treatment without interruption. The anti-spike-RBD antibody levels will be summarised descriptively at baseline and 3 and 12 weeks post-COVID-19 booster vaccination follow-up. The differences between the study arms will be estimated using a mixed effects regression model, allowing for repeated measures clustered within participants. The model will be adjusted for a randomisation factor (first line or subsequent line of therapy), and prior infection status obtained from prevaccination antinucleocapsid antibodies, as fixed effects. A treatment by time interaction will be included. The model is anticipated to use an unstructured covariance matrix, and maximum likelihood estimation. Data will be log-transformed prior to analysis, as appropriate. The adjusted mean difference between the groups will be presented, together with 95% CI and p values. The regression model will be simplified (eg, linear regression) if the planned model does not converge.

Supplemental analyses will explore the time effect between the last COVID-19 vaccination dose and the dose given in IMPROVE. The effect of non-compliance to the randomised intervention will be explored using per-protocol analyses.

The same model as for the primary outcome will be used to analyse the 12-week PVB anti-spike-RBD antibody outcome. Similar supportive analyses as planned for the primary outcome will be performed for 12 weeks PVB data. Overall, 50% and 90% neutralisation by Wuhan and VOC along with, T cell spike response by subtype (spike for Wuhan and VOC, and nucleocapsid and membrane), will be analysed in a similar manner to anti-spike RBD antibody outcome. Other secondary outcomes will be analysed using generalised linear models for binary and continuous data, as appropriate, with model adjustment as described above.

The number of SAEs will be presented by treatment arm. The proportion of participants with at least one SAE will be compared. Details of the events will be presented, together with information on the timing of the events from randomisation.

Missing data will be described with reasons given where available; the number and percentage of individuals in the missing category will be presented by treatment arm. No data will be considered spurious in the analysis since all data will be checked and cleaned before analysis.

### Timing of analysis

The final unblinded (to the study’s non-statistician investigators) statistical analysis will take place after all follow-up has been completed, and sufficient time has been allowed for data collection and cleaning. No formal interim statistical analyses are planned or have been allowed for in the study design.

The trial statisticians will have access to the final dataset. Once the study has been completed and the main finding have been published, the CI will also have access to the final study dataset.

### Patient and public involvement

People with CLL have been involved since inception of the study. Discussion with a small group (six people) of people with CLL confirmed the trial question is of considerable relevance to the CLL community and advised on study design. A Patient and Public Involvement (PPI) representative (LD) is a coapplicant on the grant, a member of the trial management group, and a non-independent member of the TSC. A patient advisory group has been convened and will meet regularly throughout the study. The role of this group is to work with the IMPROVE study team to ensure that the perspective of people living with CLL is considered during the design, conduct and reporting of the research. This will be achieved through the group helping to raise awareness of the study, reviewing documents, inputting on any issues requested by the trial team, and advising on dissemination of the study results to the CLL community.

### Trial oversight and management

The study is sponsored by the University of Birmingham. Day-to-day management of the study and all programming and statistical activities are to be undertaken by the UKCRC Registered Trials Unit-OCTRU at the University of Oxford.

## Discussion

The IMPROVE study is designed to investigate in those with CLL on BTKi therapy for more than 1 year if a 3-week pause in therapy, compared with continuing as usual with BTKi therapy, improves immune responses to COVID-19 booster vaccination. It will assess the quantitative and qualitative humoral response, to ancestral B.1 virus and the latest variant of concern. In addition, cellular immunity will be assessed through T cell responses to ancestral B.1 and omicron variant peptide pools to give a comprehensive assessment of the adapted immune response. It is generally accepted that both binding and neutralising antibodies are correlates of protection against SARS-CoV-2 symptomatic disease, and the contribution of cellular immunity can be important in protection from severe disease among those that are immunocompromised. The study will generate data that will be generalisable for patients taking one of the currently licensed BTKi in the UK and may be applicable to patients taking this therapy for other conditions, where treatment can be safely interrupted. Treatment interruption can be associated with disease flare, and as such, data outcomes of this will be collected over the course of the study in order for the risk/benefit to be assessed to inform future clinical decision-making.

As this study focuses on patients with CLL, we have chosen to use the internationally validated EORTC QLQ-CLL17.^32^ This will identify symptom burden, physical condition/fatigue and worries/fears on health and functioning and allow direct comparison with other published data. Non-blinding of the participants is a potential limitation to the findings, although concordance with study arm will be assessed through the 3-week CRF. Importantly, laboratory personnel will be blinded to the allocation for objective primary and secondary endpoints and, as such, the results should provide clarity for patients and clinicians around any beneficial impact on immunity of pausing BTKi therapy around the time of vaccination. The published results will help to inform national and international treatment guidance.

### Ethics and dissemination

This study has been approved by Leeds East Research Ethics Committee and Health Research Authority (REC Reference:22/YH/0226, IRAS: 319057). It will be publicised to research, clinical and patient communities and other important stakeholders. This paper has been produced using the Standard Protocol Items: Recommendations for Interventional Trials (SPIRIT) reporting guidelines.^36^ Once the study is completed, we aim to publish the study results in a peer-reviewed high-impact journal and present at national and international meetings to ensure maximum impact and rapid dissemination. The study’s PPI partners will advise on the content of all public facing content for dissemination and where else the study’s results should be communicated. There will be no restrictions on the publication of study findings. All authors will be required to meet the International Committee of Medical Journal Editors (ICJME) authorship requirements. Participant-level dataset and statistical code will be made available to bona-fide researchers on request from OCTRU and the CI, once the IMPROVE study findings have been published in full. The full study protocol may be accessed from the National Insitute for Health and Care Research (NIHR) website.

Details of study monitoring arrangements and a history of changes to the protocol is available in online supplemental material.

10.1136/bmjopen-2023-077946.supp1 Supplementary data

## Supplementary Material

Reviewer comments

Author's manuscript
