# Supplementary material for: Protocol for a multicentre randomised controlled trial examining the effects of temporarily pausing Bruton tyrosine kinase inhibitor therapy to coincide with SARS-CoV-2 vaccination and its impact on immune responses in patients with chronic lymphocytic leukaemia
Source: BMJ Open. 2023 Sep 28;13(9):e077946. doi: 10.1136/bmjopen-2023-077946 (PMC10546125; doi:10.1136/bmjopen-2023-077946)
Supplement: Supplementary data [file bmjopen-2023-077946supp001.pdf]

Supplementary material:

1. Monitoring

Data Monitoring Committee (DMC) A DMC comprising of a group of three independent experts including at least one statistician and one clinician, external to the study will be constituted. It will assess the progress, conduct and critical outcomes of the study and will meet regularly throughout the study at time-points agreed by the Chair of the Committee and the CI. The DMC will review the safety data generated, including all serious adverse events, and make recommendations as to whether the protocol should be amended to protect patient safety. It will report to the chair of the TSC via the trial statistician. The DMC is independent of the sponsor and will maintain a list of conflicts of interest.

Auditing: The Oxford Clinical Trials and Research Unit (OCTRU) Quality Assurance team will undertake periodic audits on behalf of the sponsor.

Study management

The Chief Investigator has overall responsibility for the study and shall oversee all study management. The data custodian will be the Chief Investigator.

Trial Steering Committee (TSC) The role of the independent TSC is to provide the overall supervision of the study. They will monitor the study's progress and conduct, and will advise on scientific credibility. The committee will consider and act, as appropriate, upon the recommendations of the DMC and ultimately carries the responsibility for deciding whether the study needs to be stopped on grounds of safety or efficacy. The committee includes independent members and members of the research team.

Trial Management Group (TMG) consists of those individuals responsible for the operational management of the study such as the chief investigator and co-investigators, Trials group

Barber VS, *et al. BMJ Open* 2023; 13:e077946. doi: 10.1136/bmjopen-2023-077946

operational lead, the trial manager and the trial statistician. Other specialities/ individuals will be invited as required for specific items/issues.

The TMG will meet usually at least once a month throughout the lifetime of the study and will:

- Supervise the conduct and progress of the study, and adherence to the study protocol;
- Assess the safety as compiled by the study team and assessed by the DMC;
- Evaluate the quality of the study data.

2. History of changes

Protocol version no.	Protocol date	Summary of key changes from previous version
1.0	18Aug2022	1st version of the protocol.
2.0	15Sep2022	Typographical errors updated and removal of remote consent
3.0	20Sep2022	Typographical errors updated and clarification of labelling on research blood samples and update of funder logo and insertion of ISRCTN allocated
4.0	08Nov2022	Reduction of text in one of the text messages due to limit of text system for 460 characters.<br>Minor changes to text messages.<br>Correction of who reply slips are returned to.<br>Addition of window for collection of extra optional samples<br>Change to timepoint of collection of vaccination history.<br>Typographical corrections
5.0	24Mar2023	Addition of aide memoire card.<br>Update to outcome measure for secondary endpoints to include influenza vaccination given during Autumn 2023.<br>Typographical corrections.<br>Addition of funder statement to front page and study duration to synopsis (requested by funder)
