# Supplementary material for: Protocol for a multicentre randomised controlled trial examining the effects of temporarily pausing Bruton tyrosine kinase inhibitor therapy to coincide with SARS-CoV-2 vaccination and its impact on immune responses in patients with chronic lymphocytic leukaemia
Source: BMJ Open. 2023 Sep 28;13(9):e077946. doi: 10.1136/bmjopen-2023-077946 (PMC10546125; doi:10.1136/bmjopen-2023-077946)
Supplement: Author's manuscript [file bmjopen-2023-077946.draft_revisions.pdf]

BMJ Open is committed to open peer review. As part of this commitment we make the peer review history of every article we publish publicly available.

When an article is published we post the peer reviewers' comments and the authors' responses online. We also post the versions of the paper that were used during peer review. These are the versions that the peer review comments apply to.

The versions of the paper that follow are the versions that were submitted during the peer review process. They are not the versions of record or the final published versions. They should not be cited or distributed as the published version of this manuscript.

BMJ Open is an open access journal and the full, final, typeset and author-corrected version of record of the manuscript is available on our site with no access controls, subscription charges or pay-per-view fees (http://bmjopen.bmj.com).

If you have any questions on BMJ Open's open peer review process please email info.bmjopen@bmj.com

BMJ Open

Multi-centre randomised controlled trial examining the effects of temporarily pausing Bruton Tyrosine Kinase inhibitor therapy to coincide with SARS-CoV-2 vaccination and its impact on immune responses in patients with Chronic Lymphocytic Leukaemia

Journal:	*BMJ Open*
Manuscript ID	bmjopen-2023-077946
Article Type:	Protocol
Date Submitted by the Author:	19-Jul-2023
Complete List of Authors:	Barber, Vicki; University of Oxford, Nuffield Department of Orthopaedics Rheumatology and Musculoskeletal Sciences, Oxford Clinical Trials Research Unit (OCTRU)<br>Peckham, Nicholas; University of Oxford, Nuffield Department of Orthopaedics Rheumatology and Musculoskeletal Sciences, Oxford Clinical Trials Research Unit (OCTRU)<br>Duley, Lelia; University of Nottingham, Nottingham Clinical Trials Unit<br>Francis, Anne; University of Oxford, Nuffield Department of Orthopaedics, Rheumatology and Musculoskeletal Science (NDORMS) and Oxford Clinical Trials Research Unit (OCTRU)<br>Abhishek, Abhishek; University of Nottingham, Academic Rheumatology<br>Moss, Paul; University of Birmingham, Institute of Immunology and Immunotherapy<br>Cook, Jonathan; University of Oxford, University of Oxford Nuffield Department of Orthopaedics Rheumatology and Musculoskeletal Sciences, Oxford Clinical Trials Research Unit (OCTRU)<br>Parry, Helen; University of Birmingham, Institute of Immunology and Immunotherapy
Keywords:	COVID-19, Clinical trials < THERAPEUTICS, HAEMATOLOGY

SCHOLARONE™
Manuscripts

Title: Multi-centre randomised controlled trial examining the effects of temporarily pausing Bruton Tyrosine Kinase inhibitor therapy to coincide with SARS-CoV-2 vaccination and its impact on immune responses in patients with Chronic Lymphocytic Leukaemia

[Short title: The IMpact of Pausing BTKi therapy and Responsiveness Of Vaccination in blood cancEr patients: a randomised controlled study – the IMPROVE study]

Authors:

Vicki S Barber, Nicholas Peckham, Lelia Duley, Anne Francis, Abhishek Abhishek, Paul Moss, Jonathan A Cook, Helen M Parry

Affiliations:

HMP, PM – Institute of Immunology and Immunotherapy, University of Birmingham, Birmingham, UK

AA - Academic Rheumatology, University of Nottingham, Nottingham, UK

JAC, VSB, NP, AF – Oxford Clinical Trials Research Unit, Nuffield Department of Orthopaedics, Rheumatology and Musculoskeletal Sciences, University of Oxford, Oxford, UK

LD – Nottingham Clinical Trials Unit, University of Nottingham, Nottingham, UK

Corresponding author: Helen Parry

Word count: 4467 words (Exc Abstract)

Abstract: 297 words

Keywords: COVID-19; clinical trials; haematology; immune response

For peer review only - http://bmjopen.bmj.com/site/about/guidelines.xhtml

Abstract:

Introduction: People who are immunocompromised have a poor biological response to vaccinations. This study aims to determine in patients with chronic lymphocytic leukaemia (CLL) if a three week pause in Bruton tyrosine kinase inhibitor therapy (BTKi) starting one week before delivery of SARS-CoV-2 vaccine booster, improves vaccine immune response when compared with continuation of BTKi.

Methods and analysis: An open label, randomised controlled superiority trial will be conducted in haematology clinics in approximately 10 UK NHS hospitals. The sample size is 120, randomised 1:1 to intervention and usual care arms. The primary outcome is anti-spike-receptor binding domain (RBD) antibody level at 3 weeks post SARS-CoV-2 booster vaccination. Secondary outcomes are RBD antibody levels at 12 weeks post-booster vaccination, participant global assessments of disease activity, blood films, full blood count and lactate dehydrogenase (LDH) levels, impact on quality of life, self-reported adherence with request to temporarily pause or continue BTKi, T cell response against spike protein and relative neutralising antibody titre against SARS-CoV-2 viral variants. Additionally, there will be an investigation of any effects in those given influenza vaccination contemporaneously versus COVID-19 alone.

The primary analysis will be performed on the as randomised groups ("intention to treat"). The difference between the study arms in anti-spike-RBD antibody level will be estimated using a mixed effects regression model, allowing for repeated measures clustered within participants. The model will be adjusted for randomisation factor (1st line or subsequent line of therapy), and prior infection status obtained from pre-randomisation anti-nucleocapsid antibodies as fixed effects.

Ethics and dissemination: This study has been approved by Leeds East Research Ethics Committee and Health Research Authority (REC Reference:22/YH/0226, IRAS ID: 319057). Dissemination will be via peer review publications, newsletters and conferences. Results will be communicated to participants, the CLL patient and clinical communities and health policymakers.

For peer review only - http://bmjopen.bmj.com/site/about/guidelines.xhtml

Trial registration: ISRCTN14197181

Protocol version (date): V5.0 24Mar2023

Article Summary

Strengths and limitations of this study:

Strengths of this study

- This study aims to recruit individuals with chronic lymphocytic leukaemia treated with BTKi therapy – which is commonly used in CLL as first and subsequent treatment but is known to negatively impact on antibody generation, to investigate if vaccination induced immune-response can be altered.
- It is adequately powered to detect modest differences in anti-spike RBD antibody titres that have been deemed would be clinically important alongside what impact there is on the functional quality of responses observed, therefore, it will provide information about both the benefits and risks of temporarily pausing treatment.
- This is an open label study so both participants and researchers will be unblinded to group allocation, however, the primary and key secondary outcome measures are laboratory assessed by staff blinded to group allocation.

Limitations of this study

- The different vaccine types available in the latest vaccination programme may impact the study results – the latest Sanofi vaccination does not contain ancestral SARS-CoV-2 spike protein which is the antigen used in the RBD spike assay upon which the primary outcome is based.

For peer review only - http://bmjopen.bmj.com/site/about/guidelines.xhtml

Introduction: Chronic Lymphocytic Leukaemia (CLL) is the most common adult leukaemia in many countries with 3,800 new diagnoses each year in the UK [1-3]. It is a chronic and currently incurable disease, that demonstrates wide clinical heterogeneity [4, 5]. CLL occurs predominantly in older people and is rare below the age of 40 years. An estimated 31,900 people are living with CLL in the UK. [6-8]

People who are immunocompromised have a poor biological response to vaccinations [9]. CLL is associated with underlying immune suppression. This can lead to considerable risk of infection with associated morbidity and mortality [10]. Infection remains the cause of death for around 25% of patients with CLL [11, 12]. Most recently, people with CLL were shown to be susceptible to severe COVID-19 and have been prioritised for receiving SARS-CoV-2 vaccines [13-17]. Unfortunately, vaccine responses in patients with CLL are impaired, particularly in those on current 'targeted' drugs (Bruton Tyrosine Kinase inhibitors (BTKi)) that act to inhibit B cell receptor function. This class of drug is highly effective in suppressing the CLL tumour and is taken continuously in the long-term [18-22]. New approaches to improve immune protection in patients with CLL are needed [23]. In this study, we seek to investigate if a pragmatic modification of CLL therapy could act to have an impact on vaccine induced immunity in this vulnerable group.

We hypothesise that people taking Bruton Tyrosine Kinase inhibitors (BTKi) specifically Ibrutinib or Acalabrutinib for their CLL at the time of vaccination against COVID-19 (+/- an influenza vaccination) will have an impaired immune response to the vaccine dose, and therefore lower production of anti-spike-receptor binding domain (RBD) and neutralising antibodies. We propose that a three-week pause in BTKi treatment will improve the vaccine-specific immune response, without significant worsening of the control of the underlying CLL, when compared to continuation of treatment as usual. The design and delivery of this study was based around another trial that assessed pausing the immune suppressant drug methotrexate around the time of COVID-19 vaccination in patients with inflammatory disorders [24]. Pausing of CLL therapy is not uncommon

For peer review only - http://bmjopen.bmj.com/site/about/guidelines.xhtml

and is recommended peri-operatively due to the off-target effect of BTKi therapy on platelet function, which results in an increased bleeding risk [25, 26]. Individuals can then experience a disease flare (which is an enlargement of the lymph nodes and B symptoms); most are mild with symptoms such as a fever, rash or mild pain, but some can experience raised lymphocyte counts, enlarged spleens, and pain and changes seen in their blood biochemical profiles.

Currently, no randomised trials have examined the impact of pausing BTKi therapy in patients with CLL for vaccine responsiveness. However, several observational studies have shown poorer responses in those taking BTKi and in addition, improvement in responses off therapy. In the CLL-VR study, following the second dose of vaccine, response rates were almost half in patients who took a BTKi compared to healthy controls [27]. Greenberger et al studied responses to a 3rd dose of COVID-19 vaccination in 25 patients with CLL, 13 of whom were treated with BTKi. Of these, 6/13 had an elevated anti-Spike response but four had stopped therapy and one was maintained on a low dose. In comparison, amongst those who continued therapy, five had a very weak response (< 5.2 U/ml) and two had an intermediate response (<250 U/ml) [28]. These data suggest that both the proportion of responders and the strength of response increases in patients not taking BTKi therapy.

Thus, the main aim of this study is to assess whether a three-week pause in BTKi treatment, 1 week before and 2 weeks after SARS-CoV-2 vaccine booster, improves the vaccine response in people with CLL, compared with continuing BTKi treatment as usual. Key secondary outcomes include any impact on quality of life and disease activity.

Objectives

Primary: Assess the effectiveness of a three-week pause in Bruton Tyrosine Kinase inhibitors (BTKi), rather than continuing treatment as usual, on anti-spike-RBD antibody levels at 3-weeks post SARS-CoV-2 booster vaccination.

For peer review only - http://bmjopen.bmj.com/site/about/guidelines.xhtml

Secondary and exploratory: Assess the effectiveness of a three-week pause in BTKi treatment rather than continuing treatment as usual, on:

- anti-spike-RBD antibody levels at week 12 after booster vaccination.
- neutralising antibody titres at baseline and week 3 after booster vaccination against Wuhan D614G and current variant of concern.
- effect on T cell response to SARS-CoV-2 spike protein at 3 weeks post-booster vaccination.
- responses to booster vaccination at 3 and 12 weeks after vaccination when influenza vaccination was given at the same time.
- biochemical disease activity at weeks 3 and 12 post-booster vaccination.
- disease activity changes and their tolerability as reported by participants during the 12 weeks post-booster vaccination.
- quality of life (QoL) at weeks 3 and 12 after booster vaccination.
- self-reported adherence to the allocation.

Methods

Study design A 2-arm parallel group, multicentre, superiority randomised controlled trial, with 1:1 randomisation. This study will be conducted in approximately 10 NHS hospitals in England and Wales. Figure 1 provides an overview of the study. These will be a mix of district general and university hospitals.

Recruitment Participants will be recruited from haematology clinics. Searching of clinic records/hospital/pharmacy databases by the usual care team and identification during routine clinic visits will be the main methods of identification of potentially eligible patients. Social media and promotion of the study by national patient charities, will enable people taking BTKi for their CLL to directly approach their local study teams (or the study centre) to find out more about the study. People identified in the database reviews will be sent a study introductory letter, a

For peer review only - http://bmjopen.bmj.com/site/about/guidelines.xhtml

participant information leaflet, and a reply slip to return if they are interested in taking part. For those identified directly in clinics, they will be offered the participant information leaflet and the study introductory letter if appropriate.

Eligibility criteria

Inclusion criteria

- Age ≥18 years.
- Confirmed diagnosis of Chronic Lymphocytic Leukaemia (CLL),
- Taking oral BTKi therapy (Acalabrutinib or Ibrutinib) for at least 12 months,
- Has achieved complete remission (including CR with incomplete marrow recovery), partial remission (including nodular partial remission (PR) or PR with lymphocytosis) or stable disease by the International Workshop on Chronic Lymphocytic Leukemia (iwCLL) response criteria [29],
- Considered able to pause BTKi therapy for three weeks without the risk of substantial increase in disease activity,
- Anticipated to take BTKi over the next 4 months,
- Willing to accept either study arm allocation,
- Able to give informed consent,
- Eligible for a planned vaccination for COVID-19.

Exclusion criteria

- Insufficient time for applying the intervention prior to the planned COVID-19 vaccination,
- Diagnosed with alternative conditions requiring treatment with BTKi,
- Treated with anti-CD20 antibody therapy in the last 18 months or planning to start it,

For peer review only - http://bmjopen.bmj.com/site/about/guidelines.xhtml

- Concurrent immune suppressive treatments in the last three months specifically: methotrexate, ciclosporin, BCL-2 inhibitors, azathioprine, mycophenolate, prednisolone and biologic agents,
- Any contraindications to COVID-19 vaccination,
- Richter's transformation requiring active therapy,
- Radiotherapy or cancer chemotherapy in last six months,
- Active solid organ cancer (people with skin cancer or those cured of solid organ cancer are eligible),
- Receiving or has received in the past 6 months immunoglobulin replacement therapy,
- Receiving or has received in the past 6 months monoclonal antibody therapy against COVID-19 spike protein.

Randomisation This will be performed using a centralised validated computer randomisation program through a secure (encrypted) web-based service, provided by the Oxford Clinical Trials Research Unit (OCTRU). Eligible participants will be randomised when they receive a date for their SARS-CoV-2 booster vaccination. The randomisation system will stratify on whether the participant is on their first line or subsequent line of therapy for CLL to ensure balanced allocation across treatment groups. It will use a 1:1 ratio to allocate to either continuing taking BTKi as usual or to have a 3-week pause (1 week before and 2 weeks after their booster vaccination against COVID-19).

Randomisation will be stratified on the line of CLL therapy being used as it has already been shown that the magnitude of immune response differs depending upon the number of lines of therapy [27]. We have chosen not to stratify on past COVID-19 infection even though it is a strong modifier of serological response to SARS-CoV-2 vaccines as it is difficult to ascertain this reliably from participant self-report. We are also not stratifying on the first vaccine platform received (e.g. adenovirus vector or mRNA) as we have previously shown that there is no difference in the

For peer review only - http://bmjopen.bmj.com/site/about/guidelines.xhtml

magnitude of antibody response by vaccine platform in patients with CLL[27], nor on which BTKi therapy the participant is currently taking. However, we will obtain past infection status using anti-nucleocapsid antibodies and use this in the statistical analysis. Due to the nature of the intervention, the participants and the clinical team will not be blind to the allocated arm of the study. However, those analysing the study samples will be blinded to the participants' allocation. Trial statisticians will not be blinded.

Treatment arms

The SARS-CoV-2 booster vaccination received whilst participating in the IMPROVE study will be delivered by the UK's national vaccination programme [30]. If another vaccine such as influenza is given at the same time, these data will be recorded.

Experimental arm: To suspend BTKi therapy for one week before and two weeks immediately after receiving the booster vaccination against COVID-19.

Control arm: To continue on the same dose and regime of BTKi therapy as usual in the week before and weeks after receiving the booster vaccination against COVID-19.

No concomitant care or interventions are prohibited in the study and the participants' clinical care team will continue to manage their condition in the usual way after the end of their participation in the study.

Automatic reminders by SMS or email will be sent to participants to encourage adherence to their randomised intervention, where they consent to receive these. Participants may be telephoned by the study team if they decline the use of SMS or emails for reminders.

Participants and their usual care team will be able to manage any potential tumour flares including with steroids or any other drug as clinically appropriate. Should a clinical need arise, the participants' usual care team will be able to advise them to continue or not with their allocation. It is known that tumour flare is fully reversible with the re-introduction of BTKi [25], and planned dose

For peer review only - http://bmjopen.bmj.com/site/about/guidelines.xhtml

suspensions are common and do not appear to compromise long term outcomes such as progression free or overall survival [31].

Outcomes

Primary outcome

- SARS-CoV-2 spike RBD-specific antibody titre at 3 weeks post booster vaccination (PBV).

Secondary outcomes

- Levels of anti-spike RBD antibody at 12 weeks PBV.
- Participant assessments of disease activity: global assessment using a numeric rating scale with one-week recall at baseline, 3 and 12 weeks PBV, current disease activity level and change since baseline, 3 and 12 weeks PBV.
- Disease flare-up (tumour flare) and actions taken to deal with them at 3 and 12 weeks PBV.
- Effect on QoL (assessed using EORTC-QLQ-CLL17) at 3 and 12 weeks PBV.
- Adherence with advice to pause or continue BTKi: self-report at 3 weeks PBV.
- COVID-19 neutralising titre at baseline and 3 weeks PBV.
- T cell responses against ancestral Wuhan and the latest variant of concern (B1, B4, B5 and Sanofi) at baseline and 3 weeks PBV.

Exploratory outcomes

- Effect on co-administration of influenza vaccination

Safety outcomes

- Serious Adverse Events (SAEs) that are related to study intervention (recorded from booster vaccination to 12 weeks PBV).

Data to be collected: Data collection will occur after informed written consent is obtained by a site principal investigator (PI) or delegated member of their research team. Participants will also be asked to decide whether to they wish to give an optional consent for storage of any outstanding

For peer review only - http://bmjopen.bmj.com/site/about/guidelines.xhtml
For peer review only

samples to a Licensed Biobank at the end of the study. Table 1 lists all of the data points and data collected through the study.

Baseline Visit: Data on demographic factors (age, sex, ethnicity, height, weight, usual residence (home or residential care)). CLL history (number of lines of therapy, which BTKi is taken and starting dates of therapies), CLL disease activity; bloods taken to measure full blood count (FBC) (haemoglobin, platelet count, lymphocyte count); blood film (presence of prolymphocytes, smear cells or lymphocytosis); and lactate dehydrogenase (LDH) levels; smoking status; self-reported physician diagnosis of comorbidities: diabetes including diet-controlled diabetes, hypertension, ischaemic heart disease, congestive cardiac failure, asthma, chronic obstructive pulmonary disease (COPD), high cholesterol, stroke including transient ischaemic attack; current medications; past SARS-CoV-2 vaccines will be collected by the local research team. QoL will be assessed using EORTC-QLQ-CLL17 [32]. Patient global assessment of disease activity will be assessed on a 0-10 numeric rating scale (NRS) for past week using the question: *"Over the LAST 7 DAYS, in all the ways that your CLL affects you, how would you rate the way you have felt?" In addition to answering yes or no on whether they have had a temperature greater than 37.5°C, and/or noticed new or enlarged lymph nodes.*

Three weeks PBV (+/- 4 days): Information about any COVID-19 positive testing, a participant's global assessment of disease activity will be collected, the latter using the same questions asked at the baseline visit asking for the last 7 days and also in the 3 week period since their booster. QoL will be assessed using EORTC-QLQ-CLL17. These data will be generally collected using a link to the study's online REDCap database sent in a text message, or by email if participant prefers not to use a mobile phone for this purpose. If a participant prefers not to receive this survey link by email or text, this information will be collected by postal questionnaires or at the study visit.

For peer review only - http://bmjopen.bmj.com/site/about/guidelines.xhtml

The local research team will collect data at this visit about a participants COVID-19 vaccination history including the most recent COVID-19 vaccination received. Details of any other vaccinations co-administered and adherence to the study intervention. In addition, details of any monoclonal antibody treatment will be collected and bloods taken and blood tests undertaken as at through taken at baseline. Details of any SAEs will also be recorded.

Week 12 PBV (+/- 7 days). Information about any COVID-19 positive testing, any influenza vaccination since the 3 week study visit, the participant's global assessment of their disease activity will be collected, the latter using the same questions asked at the baseline visit asking about the last 7 days as at Week 3. QoL will be assessed using EORTC-QLQ-CLL17 and data will be collected in the methods used at 3 weeks.

The local research team will collect data at this visit about any changes in the participants BTKi prescription, if any NHS services have been sought since the 3 week study visit, or if participants have had any course(s) of steroids since their last visit and details of any monoclonal antibody treatment will be collected. The same bloods and blood tests will be taken as at 3 weeks. Details of any SAEs will also be recorded.

Participants are free to withdraw from the study at any time and all data and samples collected up to the point of withdrawal will be used. Data will be managed and accessible as per the study's data management plan (DMP).

Sample collection and transport: Blood (10ml) will be collected in a serum activator tube at baseline, week 3 and 12 visits, along with four lithium heparin tubes (10ml). These samples will be transported to a central laboratory (the Clinical Immunology Service) at the University of Birmingham by courier. The central laboratory will centrifuge the samples on the day of arrival, aliquot in cryovials and stored at -80 degrees C. They will then be placed into dry ice storage until the analyses are undertaken. For participants recruited at two of the recruiting centres

For peer review only - http://bmjopen.bmj.com/site/about/guidelines.xhtml

(Birmingham and London), twenty participants will be sought to give optional additional blood samples, specifically the same as collected at the baseline, week 3 and 12 visits and within 48 hours and at 1 week PBV.

A further 4ml EDTA blood tube and 4ml serum separating blood tube will also be taken at the baseline, 3 and 12 week visits and processed in the local hospital laboratory to measure FBC and a blood film, and LDH levels accordingly.

Laboratory Analyses*:*

[1] Anti-spike-RBD (primary endpoint at 3 weeks) and anti-nucleocapsid antibodies: Antibody measurements will be undertaken at the University of Birmingham – Clinical Immunology Service (UoB CIS) using validated commercial assays ROCHE-S and ROCHE-N for anti-spike-RBD and anti-nucleocapsid antibodies respectively [33] [34].

ROCHE S refers to the Roche Elecsys® Anti-SARS-CoV-2 S immunoassay for the in vitro quantitative determination of antibodies (including IgG) to the SARS-CoV-2 spike protein RBD. The assay uses a recombinant protein representing the RBD of the S antigen in a double-antigen sandwich assay format, which favours detection of high affinity antibodies against SARS-CoV-2.

ROCHE N refers to the Roche Elecsys® Anti-SARS-CoV-2 assay. It uses a modified double-antigen sandwich immunoassay using recombinant nucleocapsid protein (N), which is geared towards the detection of late, mature, high affinity antibodies independent of the subclass. It is a total SARS-CoV-2 antibody assay (IgA, IgM, and IgG) detecting predominantly, but not exclusively, IgG.

[2] Neutralising antibody titres: Neutralisation assays using pseudotyped virus containing spike protein form Wuhan Hu-1 SARS-CoV-2 and a range of viral variants will be performed at the University of Glasgow on aliquots of the serum collected at Baseline and at 3 weeks. All

For peer review only - http://bmjopen.bmj.com/site/about/guidelines.xhtml

experiments will be conducted in duplicate and absorbance readings will be standardised against positive and negative controls and averaged. Neutralisation curves will be plotted, with the percentage neutralisation modelled as a logistic function of the serum dilution factor (log10). A non-linear regression (curve fit) method will be used to determine the dilution fold that neutralised 50% (IC50) of samples.

[3] T cell ELIspot analysis: T cell ELIspot analysis will be performed at the University of Birmingham to detect IFN gamma cytokine release by T cells after extraction of PBMCs from the whole blood received in the lithium heparin tube at baseline and at 3 weeks. All experiments will be conducted in duplicate and absorbance readings will be standardised against positive and negative controls and averaged.

[4] Serum immunoglobulins: Serum immunoglobulins will be analysed by the UoB CIS. Quantification of IgG, IgA and IgM will be evaluated using COBAS 6000 (Roche).

[5] Anti-flu antibodies: Serum anti-flu antibodies will be analysed by the UoB CIS. Specifically, these will be measured by ELISA for 4 H1N1 antigens to reflect the current vaccine component for those that have received a flu vaccine.

Data protection and Confidentiality: Personal information about potential and enrolled participants will be collected and processed securely, in compliance with the DPA and GDPR.

Sample size and justification

A total of 120 participants will be randomised. The sample size estimates were based on the anti-spike-RBD response after vaccination dose from a small observational study of patients with CLL who were either taking or not taking BTKi [27]. This suggested large suppressive effect of BTKi (effect size of 0.9SD). However, anti-spike RBD response related to booster vaccination in CCL patients suggests a smaller though still large effect is more plausible (circa 0.7SD). Conservatively we have based the sample size calculation upon an effect size of 0.65SD (detecting at least a 0.97 unit (log10 scale) higher antibody response in the BTKi suspension group with 90% statistical

For peer review only - http://bmjopen.bmj.com/site/about/guidelines.xhtml

power at 2-sided 5% significance level which requires data from 102 participants. This leads to 120 participants after allowing for 15% missing data. This calculation was performed using the Stata version 15.1 'power twomeans' command.

Statistical analysis Full details will be presented in a separate statistical analysis plan (SAP) which will be drafted and finalised and aimed to be published prior to the final analysis data lock and will receive review and input from the trial steering committee (TSC) and data monitoring committee (DMC). The principal analysis will be performed on the as randomised ("intention to treat") population, analysing participants with available outcome data in their randomised groups, regardless of adherence. The study will be reported in line with Consolidated Standards of Reporting Trials (CONSORT) guidelines [35].

The primary objective of the statistical analysis is to identify if a temporary 3 week pausing of BTKi around the time of vaccination against SARS-CoV-2 increases the anti-spike-RBD antibody at 3 weeks post vaccine compared to continuing BTKi treatment without interruption. The anti-spike-RBD antibody levels will be summarised descriptively at baseline and 3, and 12-weeks post COVID-19 vaccination follow-up. The differences between the study arms will be estimated using a mixed effects regression model, allowing for repeated measures clustered within participants. The model will be adjusted for the randomisation factor (1st line or subsequent line of therapy), and prior infection status obtained from pre-vaccination anti-nucleocapsid antibodies, as fixed effects. A treatment by time interaction will be included. The model is anticipated to use an unstructured covariance matrix, and maximum likelihood estimation. Data will be log-transformed prior to analysis, as appropriate. The adjusted mean difference between the groups will be presented, together with 95% CI and p-values. The regression model will be simplified (e.g. linear regression) if the planned model does not converge.

For peer review only - http://bmjopen.bmj.com/site/about/guidelines.xhtml

Supplemental analyses will explore the time effect between the last COVID-19 vaccination dose and the dose given in IMPROVE. The effect of non-compliance to the randomised intervention will be explored using per-protocol analyses.

The same model as for the primary outcome will be used to analyse the 12 week PVB anti-spike-RBD antibody outcome. Similar supportive analyses as planned for the primary outcome will be performed for 12 week PVB data. 50% and 90% neutralisation by Wuhan and VOC along with, T cell spike response by subtype (spike for Wuhan and VOC, and nucleocapsid and membrane) will be analysed in a similar manner to anti-spike RBD antibody outcome. Other secondary outcomes will be analysed using generalised linear models for binary and continuous data, as appropriate, with model adjustment as described above.

The number of SAEs will be presented by treatment arm. The proportion of participants with at least one SAE will be compared. Details of the events will be presented, together with information on the timing of the events from randomisation.

Missing data will be described with reasons given where available; the number and percentage of individuals in the missing category will be presented by treatment arm. No data will be considered spurious in the analysis since all data will be checked and cleaned before analysis.

Timing of analysis: The final unblinded (to the study's non-statistician investigators) statistical analysis will take place after all follow-up has been completed, and sufficient time has been allowed for data collection and cleaning. No formal interim statistical analyses are planned or have been allowed for in the study design.

The trial statisticians will have access to the final dataset. Once the study has been completed and the main finding have been published, the CI will also have access to the final study dataset.

Patient and public involvement

For peer review only - http://bmjopen.bmj.com/site/about/guidelines.xhtml

People with CLL have been involved since inception of the study. Discussion with a small group (6 people) of people with CLL confirmed the trial question is of considerable relevance to the CLL community and advised on study design. A PPI representative (LD) is a co-applicant on the grant, a member of the trial management group, and a non-independent member of the trial steering committee. A Patient Advisory Group has been convened and will meet regularly throughout the study. The role of this group is to work with the IMPROVE study team to ensure that the perspective of people living with CLL is considered during the design, conduct and reporting of the research. This will be achieved through the group helping to raise awareness of the trial, reviewing trial documents, inputting on any issues requested by the trial team, and advising on disseminate of the trial results to the CLL community.

Trial oversight and management

The study is sponsored by the University of Birmingham. Day to day management of the study and all programming and statistical activities are to be undertaken by the UKCRC Registered Trials Unit – Oxford Clinical Trials Research Unit (OCTRU) at the University of Oxford.

For peer review only

Discussion

The IMPROVE study is designed to investigate if a 3 week pause, compared with continuing as usual, in BTKi therapy amongst patients treated for more than 1 year, with Chronic Lymphocytic Leukaemia, will improve immune responses to COVID-19 vaccination. It will assess the quantitative and qualitative humoral response, to ancestral B.1 virus and the latest variant of concern. In addition, cellular immunity will be assessed through T cell responses to ancestral B.1 and omicron variant peptide pools to give a comprehensive assessment of the adapted immune response. It is generally accepted that both binding and neutralising antibodies are correlates of protection against SARS-CoV-2 symptomatic disease, and the contribution of cellular immunity can be important in protection from severe disease amongst immunocompromised. The study will generate data that will be generalisable for patients taking one of the currently licensed BTKi in the UK and may be applicable to patients taking this therapy for other conditions, where treatment can be safely interrupted. Treatment interruption can be associated with disease flare, and as such, data outcomes of this will be collected over the course of the study in order for the risk/benefit to be assessed to inform future clinical decision making.

As this study focuses on patients with CLL, we have chosen to use the internationally validated EORTC QLQ-CLL17 [32]. This will identify symptom burden, physical condition/fatigue, and worries/fears on health and functioning and allow direct comparison with other published data.

Non-blinding of the participants is a potential limitation to the findings, although concordance with study arm will be assessed through the 3 week CRF. Importantly, laboratory personnel will be blinded to the allocation for objective primary and secondary endpoints and, as such, the results should provide clarity for patients and clinicians around any beneficial impact on immunity of pausing BTKi therapy around the time of vaccination. The published results will help to inform national and international treatment guidance.

Ethics and Dissemination This study has been approved by Leeds East Research Ethics Committee and Health Research Authority (REC Reference:22/YH/0226, IRAS: 319057). It will be

For peer review only - http://bmjopen.bmj.com/site/about/guidelines.xhtml

publicised to research, clinical and patient communities and other important stakeholders. Once the study is completed, we aim to publish the study results in a peer-reviewed high impact journal and present at national and international meetings to ensure maximum impact and rapid dissemination. The study's PPI partners will advise on the content of all public facing content for dissemination and where else the study's results should be communicated. There will be no restrictions on the publication of study findings. All authors will be required to meet the ICJME authorship requirements. Participant level dataset and statistical code will be made available to bona-fide researchers upon request from OCTRU and the CI, once the IMPROVE study findings have been published in full. The full study protocol may be accessed from the NIHR website.

For peer review only

For peer review only - http://bmjopen.bmj.com/site/about/guidelines.xhtml

Footnotes:

Twitter: @ProfJACook, @OCTRUctu

Contributors: VSB wrote the first draft of the protocol paper. HMP is the Chief investigator of the study and conceived it. HMP, LD, AA, PM, JAC are grant holders. JAC provided statistical expertise in clinical trial design and NP is conducting the primary statistical analysis. All co-authors reviewed it critically, contributed intellectually, and approved the final version.

Funding: The study was funded by the National Institute for Health Research (NIHR) – Efficacy Mechanism Evaluation programme under the reference NIHR151892 (https://fundingawards.nihr.ac.uk/award/NIHR151892). The views expressed in this document are those of the author(s) and not necessarily those of the NIHR or the Department of Health and Social Care. The study is sponsored by The University of Birmingham, Birmingham, UK (researchgovernance@contacts.bham.ac.uk), and is being managed by the Oxford Clinical Trials Research Unit (OCTRU) at the University of Oxford. The study sponsor and funders do not have any role in study design; collection, management, analysis, and interpretation of data; writing of the report; and the decision to submit the report for publication. The study funder and sponsor do not have authority over any of these activities.

Competing interests: The institutions of all co-authors received funding from the NIHR-MRC-EME programme (NIHR151892) for conducting this research. In addition, HP reports research grants from Medical Research Council, NIHR and National Core Studies in the past 36 months and advisory fees and personal payments for lectures for AstraZeneca, Janssen and Abbvie pharmaceuticals. AA reports institutional research grants from Astra Zeneca and Oxford Immunotec, personal author royalties from UpTodate and Springer, personal consulting fees from Inflazome and NGM Biopharmaceuticals, personal payments for lectures from Menarini pharmaceuticals and Cadilla pharmaceuticals, in the previous 36 months, and unrelated to the

For peer review only - http://bmjopen.bmj.com/site/about/guidelines.xhtml

current work. AA, JAC and LD report research grants from the NIHR programmes in the last 36 months.

REFERENCES

1. CRUK. *Chronic lymphocytic leukaemia (CLL) incidence statistics* 2015 12/01/2022].
2. Hallek, M., *Chronic lymphocytic leukemia: 2017 update on diagnosis, risk stratification, and treatment.* Am J Hematol, 2017. **92**(9): p. 946-965.
3. National Cancer Institute Surveillance, E., and End Results Programme *Cancer Stat Facts: Leukemia - Chronic Lymphocytic Leukemia (CLL).* [cited 2023 19 June]; Available from: https://seer.cancer.gov/statfacts/html/clyl.html.
4. Sharma, S. and K.R. Rai, *Chronic lymphocytic leukemia (CLL) treatment: So many choices, such great options.* Cancer, 2019. **125**(9): p. 1432-1440.
5. Milne, K., B. Sturrock, and T. Chevassut, *Chronic Lymphocytic Leukaemia in 2020: the Future Has Arrived.* Curr Oncol Rep, 2020. **22**(4): p. 36.
6. Watson, L., P. Wyld, and D. Catovsky, *Disease burden of chronic lymphocytic leukaemia within the European Union.* Eur J Haematol, 2008. **81**(4): p. 253-8.
7. Molica, S., *Sex differences in incidence and outcome of chronic lymphocytic leukemia patients.* Leuk Lymphoma, 2006. **47**(8): p. 1477-80.
8. *HMRN Incidence* 08/05/2023]; Available from: hmrn.org/statistics/incidence.
9. Lee A R Y B, W.S.Y., Chai L Y A, Lee S C, Lee M X, Muthiah M D et al, *Efficacy of covid-19 vaccines in immunocompromised patients: systematic review and meta-analysis.* BMJ, 2022. **376**: p. e068632.
10. Molica, S., D. Levato, and L. Levato, *Infections in chronic lymphocytic leukemia. Analysis of incidence as a function of length of follow-up.* Haematologica, 1993. **78**(6): p. 374-7.
11. Molica, S., *Infections in chronic lymphocytic leukemia: risk factors, and impact on survival, and treatment.* Leuk Lymphoma, 1994. **13**(3-4): p. 203-14.
12. Andersen, M.A., et al., *Incidence and predictors of infection among patients prior to treatment of chronic lymphocytic leukemia: a Danish nationwide cohort study.* Haematologica, 2018. **103**.
13. Paneesha, S., et al., *Covid-19 infection in therapy-naive patients with B-cell chronic lymphocytic leukemia.* Leuk Res, 2020. **93**: p. 106366.
14. Scarfo, L., et al., *COVID-19 severity and mortality in patients with chronic lymphocytic leukemia: a joint study by ERIC, the European Research Initiative on CLL, and CLL Campus.* Leukemia, 2020. **34**(9): p. 2354-2363.
15. Mato, A.R., et al., *Outcomes of COVID-19 in patients with CLL: a multicenter international experience.* Blood, 2020. **136**(10): p. 1134-1143.
16. Noori, M., et al., *A systematic review and meta-analysis of immune response against first and second doses of SARS-CoV-2 vaccines in adult patients with hematological malignancies.* Int Immunopharmacol, 2022. **110**: p. 109046.
17. Jimenez, M., et al., *Kinetics of cellular and humoral immunogenicity and effectiveness of SARS-CoV-2 booster vaccination in hematologic neoplasms.* Am J Hematol, 2023.
18. Mauro, F.R., et al., *Response to the conjugate pneumococcal vaccine (PCV13) in patients with chronic lymphocytic leukemia (CLL).* Leukemia, 2021. **35**(3): p. 737-746.
19. Pleyer, C., et al., *Effect of Bruton tyrosine kinase inhibitor on efficacy of adjuvanted recombinant hepatitis B and zoster vaccines.* Blood, 2021. **137**(2): p. 185-189.
20. van der Velden, A.M., et al., *Influenza virus vaccination and booster in B-cell chronic lymphocytic leukaemia patients.* Eur J Intern Med, 2001. **12**(5): p. 420-424.
21. Zent, C.S., et al., *Short term results of vaccination with adjuvanted recombinant varicella zoster glycoprotein E during initial BTK inhibitor therapy for CLL or lymphoplasmacytic lymphoma.* Leukemia, 2021. **35**(6): p. 1788-1791.

For peer review only - http://bmjopen.bmj.com/site/about/guidelines.xhtml

22. Herishanu, Y., et al., *Six-month antibody persistence after BNT162b2 mRNA COVID-19 vaccination in patients with chronic lymphocytic leukemia.* Blood Adv, 2022. **6**(1): p. 148-151.
23. Moreno, C., et al., *Restoration of the immune function as a complementary strategy to treat Chronic Lymphocytic Leukemia effectively.* J Exp Clin Cancer Res, 2021. **40**(1): p. 321.
24. Abhishek, A., et al., *Effect of a 2-week interruption in methotrexate treatment versus continued treatment on COVID-19 booster vaccine immunity in adults with inflammatory conditions (VROOM study): a randomised, open label, superiority trial.* Lancet Respir Med, 2022. **10**(9): p. 840-850.
25. Hampel, P.J., et al., *Disease Flare During Temporary Interruption of Ibrutinib Therapy in Patients with Chronic Lymphocytic Leukemia.* Oncologist, 2020. **25**(11): p. 974-980.
26. Hou, J.Z., et al., *Real-world ibrutinib dose reductions, holds and discontinuations in chronic lymphocytic leukemia.* Future Oncol, 2021. **17**(35): p. 4959-4969.
27. Parry, H., et al., *Impaired neutralisation of SARS-CoV-2 delta variant in vaccinated patients with B cell chronic lymphocytic leukaemia.* J Hematol Oncol, 2022. **15**(1): p. 3.
28. Greenberger, L.M., et al., *Anti-spike antibody response to SARS-CoV-2 booster vaccination in patients with B cell-derived hematologic malignancies.* Cancer Cell, 2021. **39**(10): p. 1297-1299.
29. Hallek, M., et al., *iwCLL guidelines for diagnosis, indications for treatment, response assessment, and supportive management of CLL.* Blood, 2018. **131**(25): p. 2745-2760.
30. Agency, U.H.S., *COVID-19 vaccination programme - documents relating to the COVID-19 vaccination programme* Crown
31. Ahn, I.E., et al., *Clinically indicated ibrutinib dose interruptions and reductions do not compromise long-term outcomes in CLL.* Blood, 2019. **133**(22): p. 2452-2455.
32. Oerlemans, S., et al., *International validation of the EORTC QLQ-CLL17 questionnaire for assessment of health-related quality of life for patients with chronic lymphocytic leukaemia.* Br J Haematol, 2022. **197**(4): p. 431-441.
33. Duggan, J.O., A.; Andrews, N., *Evaluation of Roche Elecsys anti-SARS-CoV-2 S serology assay for the detection of anti-SARS-CoV-2 S antibodies*, P.H. England, Editor. 2021, Crown.
34. Group, N.S.-C.-S.A.E., *Performance characteristics of five immunoassays for SARS-CoV-2: a head-to-head benchmark comparison.* Lancet Infect Dis, 2020. **20**: p. 1390-400.
35. Schulz, K.F., et al., *CONSORT 2010 statement: updated guidelines for reporting parallel group randomised trials.* BMJ, 2010. **340**: p. c332.

For peer review only

For peer review only - http://bmjopen.bmj.com/site/about/guidelines.xhtml

Table 1: IMPROVE study research assessments at different time-points

**Assessments**	**Pre-Vaccine (baseline)**	**Vaccine date known**	**1 week before Vaccination**	**At Vaccination**	**1 week after Vaccination**	**3 weeks after Vaccination**	**12 weeks after Vaccination**
Demographics	*						
Height and weight	*						
Current medication	*						
Comorbidities	*						
Past SARS-CoV-2 vaccines	*						
Serum immunoglobins	*						
Disease activity	*					*	*
Quality of life	*					*	*
Randomisation to include a check that no monoclonal antibody has been received since baseline visit		*					
Reminders of allocation to continue or withhold BTKi			*	*	*		
Adherence to intervention						*	
Check on any monoclonal antibody (prophylaxis or treatment for COVID-19)		*				*	*
Safety						*	*
Details of vaccination						*	
Provision of aide memoire card to participant	*						
Thank you note sent after visit	*					*	*
**Samples**	**Pre-Vaccine (baseline)**	**Vaccine date known**	**1 week before Vaccination**	**At Vaccination**	**1 week after Vaccination**	**3 weeks after Vaccination**	**12 weeks after Vaccination**
Blood sample taken for anti-spike RBD antibody	*					*	*
Blood sample taken for T-cell ELISPOT	*					*	
Blood sample taken for blood film	*					*	*

For peer review only

Blood sample taken for neutralisation assay	*					*	
Blood sample taken for LDH measurement	*					*	*
Blood sample taken for full blood count measurement	*					*	*
Blood sample taken to be banked for mechanistic work as per section 11				§	§	* (no extra blood volume required from that already being taken)	* (no extra blood volume required from that already being taken)

§ To be taken from 20 participants only

For peer review only

Figure 1: Participant flow in the IMPROVE Study.

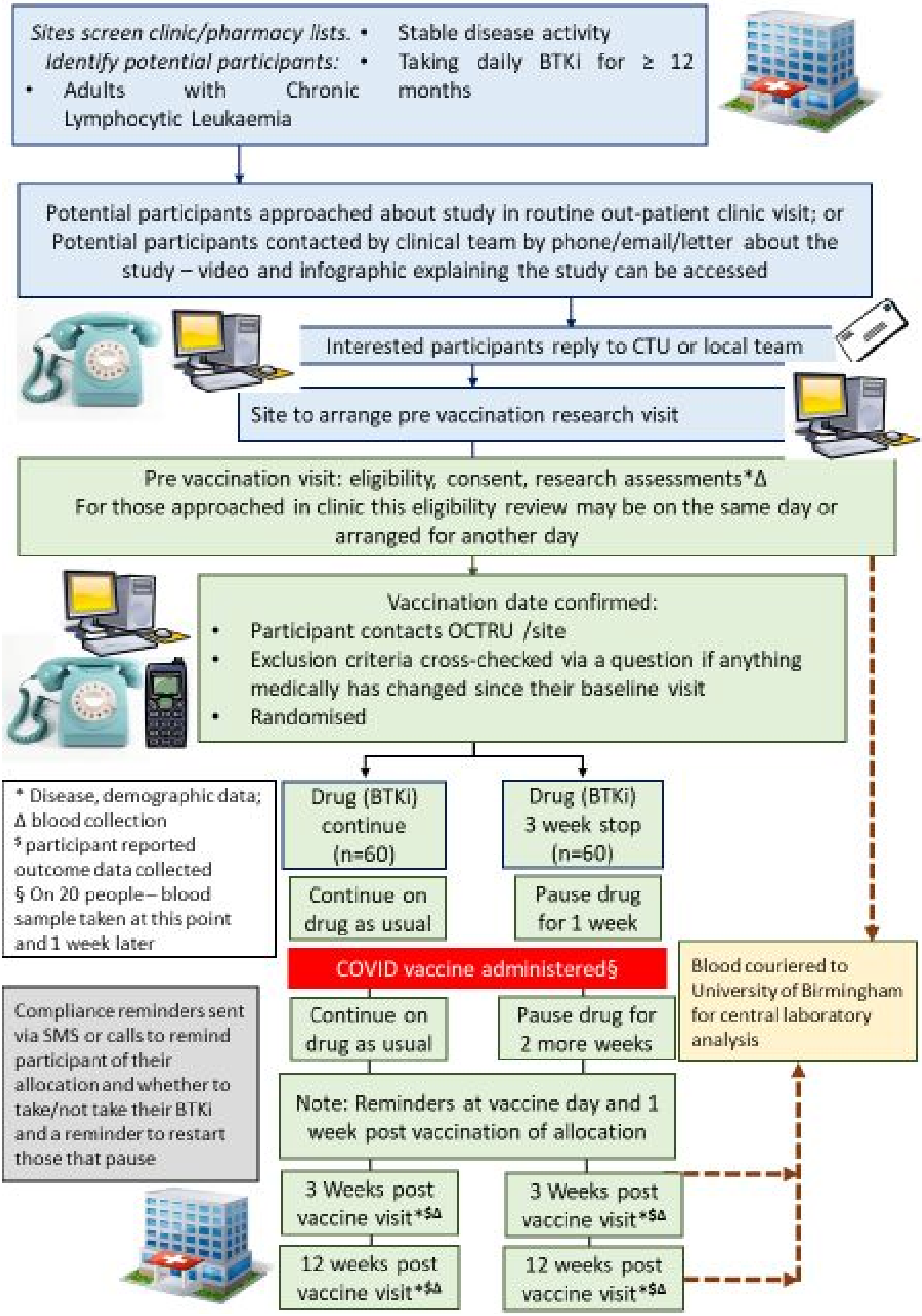

Supplementary material:

1. Monitoring

Data Monitoring Committee (DMC) A DMC comprising of a group of three independent experts including at least one statistician and one clinician, external to the study will be constituted. It will assess the progress, conduct and critical outcomes of the study and will meet regularly throughout the study at time-points agreed by the Chair of the Committee and the CI. The DMC will review the safety data generated, including all serious adverse events, and make recommendations as to whether the protocol should be amended to protect patient safety. It will report to the chair of the TSC via the trial statistician. The DMC is independent of the sponsor and will maintain a list of conflicts of interest.

Auditing: The Oxford Clinical Trials and Research Unit (OCTRU) Quality Assurance team will undertake periodic audits on behalf of the sponsor.

Study management

The Chief Investigator has overall responsibility for the study and shall oversee all study management. The data custodian will be the Chief Investigator.

Trial Steering Committee (TSC) The role of the independent TSC is to provide the overall supervision of the study. They will monitor the study's progress and conduct, and will advise on scientific credibility. The committee will consider and act, as appropriate, upon the recommendations of the DMC and ultimately carries the responsibility for deciding whether the study needs to be stopped on grounds of safety or efficacy. The committee includes independent members and members of the research team.

Trial Management Group (TMG) consists of those individuals responsible for the operational management of the study such as the chief investigator and co-investigators, Trials group operational lead, the trial manager and the trial statistician. Other specialities/ individuals will be invited as required for specific items/issues.

For peer review only - http://bmjopen.bmj.com/site/about/guidelines.xhtml

The TMG will meet usually at least once a month throughout the lifetime of the study and will:

- Supervise the conduct and progress of the study, and adherence to the study protocol;
- Assess the safety as compiled by the study team and assessed by the DMC;
- Evaluate the quality of the study data.

2. History of changes

Protocol version no.	Protocol date	Summary of key changes from previous version
1.0	18Aug2022	1st version of the protocol.
2.0	15Sep2022	Typographical errors updated and removal of remote consent
3.0	20Sep2022	Typographical errors updated and clarification of labelling on research blood samples and update of funder logo and insertion of ISRCTN allocated
4.0	08Nov2022	Reduction of text in one of the text messages due to limit of text system for 460 characters.<br>Minor changes to text messages.<br>Correction of who reply slips are returned to.<br>Addition of window for collection of extra optional samples<br>Change to timepoint of collection of vaccination history.<br>Typographical corrections
5.0	24Mar2023	Addition of aide memoire card.<br>Update to outcome measure for secondary endpoints to include influenza vaccination given during Autumn 2023.<br>Typographical corrections.<br>Addition of funder statement to front page and study duration to synopsis (requested by funder)

For peer review only

Reporting checklist for protocol of a clinical trial.

Based on the SPIRIT guidelines.

Instructions to authors

Complete this checklist by entering the page numbers from your manuscript where readers will find each of the items listed below.

Your article may not currently address all the items on the checklist. Please modify your text to include the missing information. If you are certain that an item does not apply, please write "n/a" and provide a short explanation.

Upload your completed checklist as an extra file when you submit to a journal.

In your methods section, say that you used the SPIRITreporting guidelines, and cite them as:

Chan A-W, Tetzlaff JM, Gøtzsche PC, Altman DG, Mann H, Berlin J, Dickersin K, Hróbjartsson A, Schulz KF, Parulekar WR, Krleža-Jerić K, Laupacis A, Moher D. SPIRIT 2013 Explanation and Elaboration: Guidance for protocols of clinical trials. BMJ. 2013;346:e7586

For peer review only

		Reporting Item	Page Number
**Administrative information**			
Title	#1	Descriptive title identifying the study design, population, interventions, and, if applicable, trial acronym	1
Trial registration	#2a	Trial identifier and registry name. If not yet registered,	3

		name of intended registry	
Trial registration: data set	#2b	All items from the World Health Organization Trial Registration Data Set	On ISRCTN record
Protocol version	#3	Date and version identifier	3
Funding	#4	Sources and types of financial, material, and other support	19
Roles and responsibilities: contributorship	#5a	Names, affiliations, and roles of protocol contributors	19
Roles and responsibilities: sponsor contact information	#5b	Name and contact information for the trial sponsor	19
Roles and responsibilities: sponsor and funder	#5c	Role of study sponsor and funders, if any, in study design; collection, management, analysis, and interpretation of data; writing of the report; and the decision to submit the report for publication, including whether they will have ultimate authority over any of these activities	19
Roles and responsibilities: committees	#5d	Composition, roles, and responsibilities of the coordinating centre, steering committee, endpoint adjudication committee, data management team, and	26

For peer review only

		other individuals or groups overseeing the trial, if applicable (see Item 21a for data monitoring committee)	
**Introduction**			
Background and rationale	#6a	Description of research question and justification for undertaking the trial, including summary of relevant studies (published and unpublished) examining benefits and harms for each intervention	4
Background and rationale: choice of comparators	#6b	Explanation for choice of comparators	4
Objectives	#7	Specific objectives or hypotheses	5
Trial design	#8	Description of trial design including type of trial (eg, parallel group, crossover, factorial, single group), allocation ratio, and framework (eg, superiority, equivalence, non-inferiority, exploratory)	6
**Methods: Participants, interventions, and outcomes**			
Study setting	#9	Description of study settings (eg, community clinic, academic hospital) and list of countries where data will be collected. Reference to where list of study sites can be obtained	6

For peer review only

Eligibility criteria	#10	Inclusion and exclusion criteria for participants. If applicable, eligibility criteria for study centres and individuals who will perform the interventions (eg, surgeons, psychotherapists)	7
Interventions: description	#11a	Interventions for each group with sufficient detail to allow replication, including how and when they will be administered	9
Interventions: modifications	#11b	Criteria for discontinuing or modifying allocated interventions for a given trial participant (eg, drug dose change in response to harms, participant request, or improving / worsening disease)	9
Interventions: adherance	#11c	Strategies to improve adherence to intervention protocols, and any procedures for monitoring adherence (eg, drug tablet return; laboratory tests)	9 and 12
Interventions: concomitant care	#11d	Relevant concomitant care and interventions that are permitted or prohibited during the trial	12
Outcomes	#12	Primary, secondary, and other outcomes, including the specific measurement variable (eg, systolic blood pressure), analysis metric (eg, change from baseline, final value, time to event), method of aggregation (eg, median, proportion), and time point for each outcome. Explanation of the clinical relevance of chosen efficacy and harm outcomes is strongly recommended	10

For peer review only

Participant timeline	#13	Time schedule of enrolment, interventions (including any run-ins and washouts), assessments, and visits for participants. A schematic diagram is highly recommended (see Figure)	11 and 25
Sample size	#14	Estimated number of participants needed to achieve study objectives and how it was determined, including clinical and statistical assumptions supporting any sample size calculations	14
Recruitment	#15	Strategies for achieving adequate participant enrolment to reach target sample size	6
**Methods: Assignment of interventions (for controlled trials)**			
Allocation: sequence generation	#16a	Method of generating the allocation sequence (eg, computer-generated random numbers), and list of any factors for stratification. To reduce predictability of a random sequence, details of any planned restriction (eg, blocking) should be provided in a separate document that is unavailable to those who enrol participants or assign interventions	8
Allocation concealment mechanism	#16b	Mechanism of implementing the allocation sequence (eg, central telephone; sequentially numbered, opaque, sealed envelopes), describing any steps to conceal the	8

For peer review only

		sequence until interventions are assigned	
Allocation: implementation	#16c	Who will generate the allocation sequence, who will enrol participants, and who will assign participants to interventions	8
Blinding (masking)	#17a	Who will be blinded after assignment to interventions (eg, trial participants, care providers, outcome assessors, data analysts), and how	9
Blinding (masking): emergency unblinding	#17b	If blinded, circumstances under which unblinding is permissible, and procedure for revealing a participant's allocated intervention during the trial	N/A
**Methods: Data collection, management, and analysis**			
Data collection plan	#18a	Plans for assessment and collection of outcome, baseline, and other trial data, including any related processes to promote data quality (eg, duplicate measurements, training of assessors) and a description of study instruments (eg, questionnaires, laboratory tests) along with their reliability and validity, if known. Reference to where data collection forms can be found, if not in the protocol	11
Data collection plan: retention	#18b	Plans to promote participant retention and complete follow-up, including list of any outcome data to be	9 and 12

For peer review only

		collected for participants who discontinue or deviate from intervention protocols	
Data management	#19	Plans for data entry, coding, security, and storage, including any related processes to promote data quality (eg, double data entry; range checks for data values). Reference to where details of data management procedures can be found, if not in the protocol	12
Statistics: outcomes	#20a	Statistical methods for analysing primary and secondary outcomes. Reference to where other details of the statistical analysis plan can be found, if not in the protocol	15
Statistics: additional analyses	#20b	Methods for any additional analyses (eg, subgroup and adjusted analyses)	16
Statistics: analysis population and missing data	#20c	Definition of analysis population relating to protocol non-adherence (eg, as randomised analysis), and any statistical methods to handle missing data (eg, multiple imputation)	15
**Methods: Monitoring**			
Data monitoring: formal committee	#21a	Composition of data monitoring committee (DMC); summary of its role and reporting structure; statement of whether it is independent from the sponsor and competing interests; and reference to where further details about its charter can be found, if not in the protocol. Alternatively, an explanation of why a DMC is	26

For peer review only

		not needed	
Data monitoring: interim analysis	#21b	Description of any interim analyses and stopping guidelines, including who will have access to these interim results and make the final decision to terminate the trial	17
Harms	#22	Plans for collecting, assessing, reporting, and managing solicited and spontaneously reported adverse events and other unintended effects of trial interventions or trial conduct	12 and 16
Auditing	#23	Frequency and procedures for auditing trial conduct, if any, and whether the process will be independent from investigators and the sponsor	N/A
**Ethics and dissemination**			
Research ethics approval	#24	Plans for seeking research ethics committee / institutional review board (REC / IRB) approval	2
Protocol amendments	#25	Plans for communicating important protocol modifications (eg, changes to eligibility criteria, outcomes, analyses) to relevant parties (eg, investigators, REC / IRBs, trial participants, trial registries, journals, regulators)	27
Consent or assent	#26a	Who will obtain informed consent or assent from potential trial participants or authorised surrogates, and	11

For peer review only

		how (see Item 32)	
Consent or assent: ancillary studies	#26b	Additional consent provisions for collection and use of participant data and biological specimens in ancillary studies, if applicable	11
Confidentiality	#27	How personal information about potential and enrolled participants will be collected, shared, and maintained in order to protect confidentiality before, during, and after the trial	14
Declaration of interests	#28	Financial and other competing interests for principal investigators for the overall trial and each study site	20 (for central trial team)
Data access	#29	Statement of who will have access to the final trial dataset, and disclosure of contractual agreements that limit such access for investigators	12
Ancillary and post trial care	#30	Provisions, if any, for ancillary and post-trial care, and for compensation to those who suffer harm from trial participation	N/A
Dissemination policy: trial results	#31a	Plans for investigators and sponsor to communicate trial results to participants, healthcare professionals, the public, and other relevant groups (eg, via publication, reporting in results databases, or other data sharing arrangements), including any publication restrictions	19
Dissemination policy:	#31b	Authorship eligibility guidelines and any intended use of	19

For peer review only

authorship		professional writers	
Dissemination policy: reproducible research	#31c	Plans, if any, for granting public access to the full protocol, participant-level dataset, and statistical code	19

Appendices

Informed consent materials	#32	Model consent form and other related documentation given to participants and authorised surrogates	Available from the trial team
Biological specimens	#33	Plans for collection, laboratory evaluation, and storage of biological specimens for genetic or molecular analysis in the current trial and for future use in ancillary studies, if applicable	13 and 14

None The SPIRIT Explanation and Elaboration paper is distributed under the terms of the Creative Commons Attribution License CC-BY-NC. This checklist can be completed online using https://www.goodreports.org/, a tool made by the EQUATOR Network in collaboration with Penelope.ai

For peer review only

BMJ Open

Protocol for a multi-centre randomised controlled trial examining the effects of temporarily pausing Bruton Tyrosine Kinase inhibitor therapy to coincide with SARS-CoV-2 vaccination and its impact on immune responses in patients with Chronic Lymphocytic Leukaemia

Journal:	*BMJ Open*
Manuscript ID	bmjopen-2023-077946.R1
Article Type:	Protocol
Date Submitted by the Author:	04-Aug-2023

Complete List of Authors:	Barber, Vicki; University of Oxford, Nuffield Department of Orthopaedics Rheumatology and Musculoskeletal Sciences, Oxford Clinical Trials Research Unit (OCTRU)<br>Peckham, Nicholas; University of Oxford, Nuffield Department of Orthopaedics Rheumatology and Musculoskeletal Sciences, Oxford Clinical Trials Research Unit (OCTRU)<br>Duley, Lelia; University of Nottingham, Nottingham Clinical Trials Unit<br>Francis, Anne; University of Oxford, Nuffield Department of Orthopaedics, Rheumatology and Musculoskeletal Science (NDORMS) and Oxford Clinical Trials Research Unit (OCTRU)<br>Abhishek, Abhishek; University of Nottingham, Academic Rheumatology<br>Moss, Paul; University of Birmingham, Institute of Immunology and Immunotherapy<br>Cook, Jonathan; University of Oxford, University of Oxford Nuffield Department of Orthopaedics Rheumatology and Musculoskeletal Sciences, Oxford Clinical Trials Research Unit (OCTRU)<br>Parry, Helen; University of Birmingham, Institute of Immunology and Immunotherapy

**Primary Subject Heading**:	Haematology (incl blood transfusion)
Secondary Subject Heading:	Medical management
Keywords:	COVID-19, Clinical trials < THERAPEUTICS, HAEMATOLOGY

SCHOLARONE™
Manuscripts

Title: Protocol for a multi-centre randomised controlled trial examining the effects of temporarily pausing Bruton Tyrosine Kinase inhibitor therapy to coincide with SARS-CoV-2 vaccination and its impact on immune responses in patients with Chronic Lymphocytic Leukaemia

[Short title: The IMpact of Pausing BTKi therapy and Responsiveness Of Vaccination in blood cancEr patients: a randomised controlled study – the IMPROVE study protocol paper]

Authors:

Vicki S Barber, Nicholas Peckham, Lelia Duley, Anne Francis, Abhishek Abhishek, Paul Moss, Jonathan A Cook, Helen M Parry

Affiliations:

HMP, PM – Institute of Immunology and Immunotherapy, University of Birmingham, Birmingham, UK

AA - Academic Rheumatology, University of Nottingham, Nottingham, UK

JAC, VSB, NP, AF – Oxford Clinical Trials Research Unit, Nuffield Department of Orthopaedics, Rheumatology and Musculoskeletal Sciences, University of Oxford, Oxford, UK

LD – Nottingham Clinical Trials Unit, University of Nottingham, Nottingham, UK

Corresponding author: Helen Parry, h.m.parry@bham.ac.uk

Word count: 4784 words (Exc Abstract)

Abstract: 305 words

Keywords: COVID-19; clinical trials; haematology; immune response

For peer review only - http://bmjopen.bmj.com/site/about/guidelines.xhtml

Abstract:

Introduction: People who are immunocompromised have a poor biological response to vaccinations. This study aims to determine in patients with chronic lymphocytic leukaemia (CLL) if a three week pause in Bruton tyrosine kinase inhibitor therapy (BTKi) starting one week before delivery of SARS-CoV-2 vaccine booster, improves vaccine immune response when compared with continuation of BTKi.

Methods and analysis: An open label, randomised controlled superiority trial will be conducted in haematology clinics in approximately 10 UK NHS hospitals. The sample size is 120, randomised 1:1 to intervention and usual care arms. The primary outcome is anti-spike-receptor binding domain (RBD) antibody level at 3 weeks post SARS-CoV-2 booster vaccination. Secondary outcomes are RBD antibody levels at 12 weeks post-booster vaccination, participant global assessments of disease activity, blood films, full blood count and lactate dehydrogenase (LDH) levels, impact on quality of life, self-reported adherence with request to temporarily pause or continue BTKi, T cell response against spike protein and relative neutralising antibody titre against SARS-CoV-2 viral variants. Additionally, there will be an investigation of any effects in those given influenza vaccination contemporaneously versus COVID-19 alone.

The primary analysis will be performed on the as randomised groups (“intention to treat”). The difference between the study arms in anti-spike-RBD antibody level will be estimated using a mixed effects regression model, allowing for repeated measures clustered within participants. The model will be adjusted for randomisation factor (first line or subsequent line of therapy), and prior infection status obtained from pre-randomisation anti-nucleocapsid antibodies as fixed effects.

Ethics and dissemination: This study has been approved by Leeds East Research Ethics Committee and Health Research Authority (REC Reference:22/YH/0226, IRAS ID: 319057). Dissemination will be via peer review publications, newsletters and conferences. Results will be communicated to participants, the CLL patient and clinical communities and health policymakers.

For peer review only - http://bmjopen.bmj.com/site/about/guidelines.xhtml

Trial registration: ISRCTN14197181

Protocol version (date): V5.0 24Mar2023

Article Summary

Strengths and limitations of this study:

Strengths of this study

- It is adequately powered to detect modest differences in anti-spike RBD antibody titres that have been deemed would be clinically important alongside what impact there is on the functional quality of responses observed, therefore, it will provide information about both the benefits and risks of temporarily pausing treatment.
- This is an open label study so both participants and researchers will be unblinded to group allocation, however, the primary and key secondary outcome measures are laboratory assessed by staff blinded to group allocation.

Limitations of this study

- The different vaccine types available in the latest vaccination programme may impact the study results – the latest Sanofi vaccination does not contain ancestral SARS-CoV-2 spike protein which is the antigen used in the RBD spike assay upon which the primary outcome is based.

For peer review only - http://bmjopen.bmj.com/site/about/guidelines.xhtml

Introduction: Chronic Lymphocytic Leukaemia (CLL) is the most common adult leukaemia in many countries with 3,800 new diagnoses each year in the UK [1-3]. It is a chronic and currently incurable disease, that demonstrates wide clinical heterogeneity [4, 5]. CLL occurs predominantly in older people and is rare below the age of 40 years. An estimated 31,900 people are living with CLL in the UK. [6-8]

People who are immunocompromised have a poor biological response to vaccinations [9]. CLL is associated with underlying immune suppression. This can lead to considerable risk of infection with associated morbidity and mortality [10]. Infection remains the cause of death for around 25% of patients with CLL [11, 12]. Most recently, people with CLL were shown to be susceptible to severe COVID-19 and have been prioritised for receiving SARS-CoV-2 vaccines [13-17]. Unfortunately, vaccine responses in patients with CLL are impaired, particularly in those on current 'targeted' drugs (Bruton Tyrosine Kinase inhibitors (BTKi)) that act to inhibit B cell receptor function. This class of drug is highly effective in suppressing the CLL tumour and is taken continuously in the long-term [18-22]. New approaches to improve immune protection in patients with CLL are needed [23]. In this study, we seek to investigate if a pragmatic modification of CLL therapy could act to have an impact on vaccine induced immunity in this vulnerable group.

We hypothesise that people taking Bruton Tyrosine Kinase inhibitors (BTKi) specifically Ibrutinib or Acalabrutinib for their CLL at the time of vaccination against COVID-19 (+/- an influenza vaccination) will have an impaired immune response to the vaccine dose, and therefore lower production of anti-spike-receptor binding domain (RBD) and neutralising antibodies. We propose that a three-week pause in BTKi treatment will improve the vaccine-specific immune response, without significant worsening of the control of the underlying CLL, when compared to continuation of treatment as usual. The design and delivery of this study was based around another trial that assessed pausing the immune suppressant drug methotrexate around the time of COVID-19 vaccination in patients with inflammatory disorders [24]. Pausing of CLL therapy is not uncommon

For peer review only - http://bmjopen.bmj.com/site/about/guidelines.xhtml

and is recommended peri-operatively due to the off-target effect of BTKi therapy on platelet function, which results in an increased bleeding risk [25, 26]. Individuals can then experience a disease flare (which is an enlargement of the lymph nodes and B symptoms); most are mild with symptoms such as a fever, rash or mild pain, but some can experience raised lymphocyte counts, enlarged spleens, and pain and changes seen in their blood biochemical profiles.

Currently, no randomised trials have examined the impact of pausing BTKi therapy in patients with CLL for vaccine responsiveness. However, several observational studies have shown poorer responses in those taking BTKi and in addition, improvement in responses off therapy. In the CLL-VR study, following the second dose of vaccine, response rates were almost half in patients who took a BTKi compared to healthy controls [27]. Greenberger et al studied responses to a third dose of COVID-19 vaccination in 25 patients with CLL, 13 of whom were treated with BTKi. Of these, 6/13 had an elevated anti-Spike response but four had stopped therapy and one was maintained on a low dose. In comparison, amongst those who continued therapy, five had a very weak response (< 5.2 U/ml) and two had an intermediate response (<250 U/ml) [28]. These data suggest that both the proportion of responders and the strength of response increases in patients not taking BTKi therapy.

Thus, the main aim of this study is to assess whether a three-week pause in BTKi treatment, 1 week before and 2 weeks after SARS-CoV-2 vaccine booster, improves the vaccine response in people with CLL, compared with continuing BTKi treatment as usual. Key secondary outcomes include any impact on quality of life and disease activity.

Objectives

Primary: Assess the effectiveness of a three-week pause in Bruton Tyrosine Kinase inhibitors (BTKi), rather than continuing treatment as usual, on anti-spike-RBD antibody levels at 3-weeks post SARS-CoV-2 booster vaccination.

For peer review only - http://bmjopen.bmj.com/site/about/guidelines.xhtml

Secondary and exploratory: Assess the effectiveness of a three-week pause in BTKi treatment rather than continuing treatment as usual, on:

- anti-spike-RBD antibody levels at week 12 after booster vaccination.
- neutralising antibody titres at baseline and week 3 after booster vaccination against Wuhan D614G and current variant of concern.
- effect on T cell response to SARS-CoV-2 spike protein at 3 weeks post-booster vaccination.
- responses to booster vaccination at 3 and 12 weeks after vaccination when influenza vaccination was given at the same time.
- biochemical disease activity at weeks 3 and 12 post-booster vaccination.
- disease activity changes and their tolerability as reported by participants during the 12 weeks post-booster vaccination.
- quality of life (QoL) at weeks 3 and 12 after booster vaccination.
- self-reported adherence to the allocation.

Methods

Study design A 2-arm parallel group, multicentre, superiority randomised controlled trial, with 1:1 randomisation. This study will be conducted in approximately 10 NHS hospitals in England and Wales. These will be a mix of district general and university hospitals. Figure 1 provides an overview of the study.

Recruitment Participants will be recruited from haematology clinics. Searching of clinic records/hospital/pharmacy databases by the usual care team and identification during routine clinic visits will be the main methods of identification of potentially eligible patients. Social media and promotion of the study by national patient charities, will enable people taking BTKi for their CLL to directly approach their local study teams (or the study centre) to find out more about the study. People identified in the database reviews will be sent a study introductory letter, a

For peer review only

participant information leaflet, and a reply slip to return if they are interested in taking part. For those identified directly in clinics, they will be offered the participant information leaflet and the study introductory letter if appropriate.

Eligibility criteria

Inclusion criteria

- Age ≥18 years.
- Confirmed diagnosis of Chronic Lymphocytic Leukaemia (CLL),
- Taking oral BTKi therapy (Acalabrutinib or Ibrutinib) for at least 12 months,
- Has achieved complete remission (including CR with incomplete marrow recovery), partial remission (including nodular partial remission (PR) or PR with lymphocytosis) or stable disease by the International Workshop on Chronic Lymphocytic Leukemia (iwCLL) response criteria [29],
- Considered able to pause BTKi therapy for three weeks without the risk of substantial increase in disease activity,
- Anticipated to take BTKi over the next 4 months,
- Willing to accept either study arm allocation,
- Able to give informed consent,
- Eligible for a planned booster vaccination for COVID-19.

Exclusion criteria

- Insufficient time for applying the intervention prior to the planned COVID-19 booster vaccination,
- Diagnosed with alternative conditions requiring treatment with BTKi,
- Treated with anti-CD20 antibody therapy in the last 18 months or planning to start it,

For peer review only

- Concurrent immune suppressive treatments in the last three months specifically: methotrexate, ciclosporin, BCL-2 inhibitors, azathioprine, mycophenolate, prednisolone and biologic agents,
- Any contraindications to COVID-19 vaccination,
- Richter's transformation requiring active therapy,
- Radiotherapy or cancer chemotherapy in last six months,
- Active solid organ cancer (people with skin cancer or those cured of solid organ cancer are eligible),
- Receiving or has received in the past 6 months immunoglobulin replacement therapy,
- Receiving or has received in the past 6 months monoclonal antibody therapy against COVID-19 spike protein.

Randomisation This will be performed using a centralised validated computer randomisation program through a secure (encrypted) web-based service, provided by the Oxford Clinical Trials Research Unit (OCTRU). Eligible participants will be randomised when they receive a date for their SARS-CoV-2 booster vaccination. The randomisation system will stratify on whether the participant is on their first line or subsequent line of therapy for CLL to ensure balanced allocation across treatment groups. It will use a 1:1 ratio to allocate to either continuing taking BTKi as usual or to have a 3-week pause (1 week before and 2 weeks after their booster vaccination against COVID-19).

Randomisation will be stratified on the line of CLL therapy being used as it has already been shown that the magnitude of immune response differs depending upon the number of lines of therapy [27]. We have chosen not to stratify on past COVID-19 infection even though it is a strong modifier of serological response to SARS-CoV-2 vaccines as it is difficult to ascertain this reliably from participant self-report. We are also not stratifying on the first vaccine platform received (e.g. adenovirus vector or mRNA) as we have previously shown that there is no difference in the

For peer review only - http://bmjopen.bmj.com/site/about/guidelines.xhtml

magnitude of antibody response by vaccine platform in patients with CLL[27], nor on which BTKi therapy the participant is currently taking. However, we will obtain past infection status using anti-nucleocapsid antibodies and use this in the statistical analysis. Due to the nature of the intervention, the participants and the clinical team will not be blind to the allocated arm of the study. However, those analysing the study samples will be blinded to the participants' allocation. Trial statisticians will not be blinded.

Treatment arms

The SARS-CoV-2 booster vaccination received whilst participating in the IMPROVE study will be delivered by the UK's national vaccination programme [30]. If another vaccine such as influenza is given at the same time, these data will be recorded.

Experimental arm: To suspend BTKi therapy for one week before and two weeks immediately after receiving the booster vaccination against COVID-19.

Control arm: To continue the same dose and regime of BTKi therapy as usual in the week before and weeks after receiving the booster vaccination against COVID-19.

No concomitant care or interventions are prohibited in the study and the participants' clinical care team will continue to manage their condition in the usual way after the end of their participation in the study.

Automatic reminders by SMS or email will be sent to participants to encourage adherence to their randomised intervention, where they consent to receive these. Participants may be telephoned by the study team if they decline the use of SMS or emails for reminders.

Participants and their usual care team will be able to manage any potential tumour flares including with steroids or any other drug as clinically appropriate. Should a clinical need arise, the participants' usual care team will be able to advise them to continue or not with their allocation. It is known that tumour flare is fully reversible with the re-introduction of BTKi [25], and planned dose

For peer review only - http://bmjopen.bmj.com/site/about/guidelines.xhtml

suspensions are common and do not appear to compromise long term outcomes such as progression free or overall survival [31].

Outcomes

Primary outcome

- SARS-CoV-2 spike RBD-specific antibody titre at 3 weeks post booster vaccination (PBV).

Secondary outcomes

- Levels of anti-spike RBD antibody at 12 weeks PBV.
- Participant assessments of disease activity: global assessment using a numeric rating scale with one-week recall at baseline, 3 and 12 weeks PBV, current disease activity level and change since baseline, 3 and 12 weeks PBV.
- Disease flare-up (tumour flare) and actions taken to deal with them at 3 and 12 weeks PBV.
- Effect on QoL (assessed using EORTC-QLQ-CLL17) at 3 and 12 weeks PBV.
- Adherence with advice to pause or continue BTKi: self-report at 3 weeks PBV.
- COVID-19 neutralising titre at baseline and 3 weeks PBV.
- T cell responses against ancestral Wuhan and the latest variant of concern (B1, B4, B5 and Sanofi) at baseline and 3 weeks PBV.

Exploratory outcomes

- Effect on co-administration of influenza vaccination

Safety outcomes

- Serious Adverse Events (SAEs) that are related to study intervention (recorded from booster vaccination to 12 weeks PBV).

Data to be collected: Data collection will occur after informed written consent is obtained by a site principal investigator (PI) or delegated member of their research team. Participants will also be asked to decide whether to they wish to give an optional consent for storage of any outstanding

For peer review only - http://bmjopen.bmj.com/site/about/guidelines.xhtml

samples to a Licensed Biobank at the end of the study. Table 1 lists all of the data points and data collected through the study.

Baseline Visit: Data on demographic factors (age, sex, ethnicity, height, weight, usual residence (home or residential care)). CLL history (number of lines of therapy, which BTKi is taken and starting dates of therapies), CLL disease activity; bloods taken to measure full blood count (FBC) (haemoglobin, platelet count, lymphocyte count); blood film (presence of prolymphocytes, smear cells or lymphocytosis); and lactate dehydrogenase (LDH) levels; smoking status; self-reported physician diagnosis of comorbidities: diabetes including diet-controlled diabetes, hypertension, ischaemic heart disease, congestive cardiac failure, asthma, chronic obstructive pulmonary disease (COPD), high cholesterol, stroke including transient ischaemic attack; current medications; past SARS-CoV-2 vaccines will be collected by the local research team. QoL will be assessed using EORTC-QLQ-CLL17 [32]. Patient global assessment of disease activity will be assessed on a 0-10 numeric rating scale (NRS) for past week using the question: *"Over the LAST 7 DAYS, in all the ways that your CLL affects you, how would you rate the way you have felt?" In addition to answering yes or no on whether they have had a temperature greater than 37.5°C, and/or noticed new or enlarged lymph nodes.*

Three weeks PBV (+/- 4 days): Information about any COVID-19 positive testing, a participant's global assessment of disease activity will be collected, the latter using the same questions asked at the baseline visit asking for the last 7 days and also in the 3 week period since their booster. QoL will be assessed using EORTC-QLQ-CLL17. These data will be generally collected using a link to the study's online REDCap database sent in a text message, or by email if participant prefers not to use a mobile phone for this purpose. If a participant prefers not to receive this survey link by email or text, this information will be collected by postal questionnaires or at the study visit.

For peer review only - http://bmjopen.bmj.com/site/about/guidelines.xhtml

The local research team will collect data at this visit about a participants COVID-19 vaccination history including the most recent COVID-19 vaccination received, details of any other vaccinations co-administered and adherence to the study intervention. In addition, details of any monoclonal antibody treatment will be collected and bloods taken and blood tests undertaken as at baseline. Details of any SAEs will also be recorded.

Week 12 PBV (+/- 7 days). Information about any COVID-19 positive testing, any influenza vaccination since the 3 week study visit, the participant's global assessment of their disease activity will be collected, the latter using the same questions asked at the baseline visit asking about the last 7 days as at Week 3. QoL will be assessed using EORTC-QLQ-CLL17 and data will be collected in the methods used at 3 weeks.

The local research team will collect data at this visit about any changes in the participants BTKi prescription, if any NHS services have been sought since the 3 week study visit, or if participants have had any course(s) of steroids since their last visit and details of any monoclonal antibody treatment will be collected. The same bloods and blood tests will be taken as at 3 weeks. Details of any SAEs will also be recorded.

Participants are free to withdraw from the study at any time and all data and samples collected up to the point of withdrawal will be used. Data will be managed and accessible as per the study's data management plan (DMP).

Sample collection and transport: Blood (10ml) will be collected in a serum activator tube at baseline, week 3 and 12 visits, along with four lithium heparin tubes (10ml). These samples will be transported to a central laboratory (the Clinical Immunology Service) at the University of Birmingham by courier. The central laboratory will centrifuge the samples on the day of arrival, aliquot in cryovials and stored at -80 degrees C. They will then be placed into dry ice storage until the analyses are undertaken. For participants recruited at two of the recruiting centres

For peer review only - http://bmjopen.bmj.com/site/about/guidelines.xhtml

(Birmingham and London), twenty participants will be sought to give optional additional blood samples, specifically the same as collected at the baseline, week 3 and 12 visits, within 48 hours and at 1 week PBV.

A further 4ml EDTA blood tube and 4ml serum separating blood tube will also be taken at the baseline, 3 and 12 week visits and processed in the local hospital laboratory to measure FBC and a blood film, and LDH levels accordingly.

Laboratory Analyses*:*

[1] Anti-spike-RBD (primary endpoint at 3 weeks) and anti-nucleocapsid antibodies: Antibody measurements will be undertaken at the University of Birmingham – Clinical Immunology Service (UoB CIS) using validated commercial assays ROCHE-S and ROCHE-N for anti-spike-RBD and anti-nucleocapsid antibodies respectively [33] [34].

ROCHE S refers to the Roche Elecsys® Anti-SARS-CoV-2 S immunoassay for the *in vitro* quantitative determination of antibodies (including IgG) to the SARS-CoV-2 spike protein RBD. The assay uses a recombinant protein representing the RBD of the S antigen in a double-antigen sandwich assay format, which favours detection of high affinity antibodies against SARS-CoV-2.

ROCHE N refers to the Roche Elecsys® Anti-SARS-CoV-2 assay. It uses a modified double-antigen sandwich immunoassay using recombinant nucleocapsid protein (N), which is geared towards the detection of late, mature, high affinity antibodies independent of the subclass. It is a total SARS-CoV-2 antibody assay (IgA, IgM, and IgG) detecting predominantly, but not exclusively, IgG.

[2] Neutralising antibody titres: Neutralisation assays using pseudotyped virus containing spike protein form Wuhan Hu-1 SARS-CoV-2 and a range of viral variants will be performed at the University of Glasgow on aliquots of the serum collected at Baseline and at 3 weeks. All

For peer review only - http://bmjopen.bmj.com/site/about/guidelines.xhtml

experiments will be conducted in duplicate and absorbance readings will be standardised against positive and negative controls and averaged. Neutralisation curves will be plotted, with the percentage neutralisation modelled as a logistic function of the serum dilution factor (log10). A non-linear regression (curve fit) method will be used to determine the dilution fold that neutralised 50% (IC50) of samples.

[3] T cell ELIspot analysis: T cell ELIspot analysis will be performed at the University of Birmingham to detect IFN gamma cytokine release by T cells after extraction of PBMCs from the whole blood received in the lithium heparin tube at baseline and at 3 weeks. All experiments will be conducted in duplicate and absorbance readings will be standardised against positive and negative controls and averaged.

[4] Serum immunoglobulins: Serum immunoglobulins will be analysed by the UoB CIS. Quantification of IgG, IgA and IgM will be evaluated using COBAS 6000 (Roche).

[5] Anti-flu antibodies: Serum anti-flu antibodies will be analysed by the UoB CIS. Specifically, these will be measured by ELISA for 4 H1N1 antigens to reflect the current vaccine component for those that have received a flu vaccine.

Data protection and Confidentiality: Personal information about potential and enrolled participants will be collected and processed securely, in compliance with the DPA and GDPR.

Sample size and justification

A total of 120 participants will be randomised. The sample size estimates were based on the anti-spike-RBD response after vaccination dose from a small observational study of patients with CLL who were either taking or not taking BTKi [27]. This suggested a large suppressive effect of BTKi (effect size of 0.9SD). However, anti-spike RBD response related to booster vaccination in CLL patients suggests a smaller though still large effect is more plausible (circa 0.7SD). Conservatively we have based the sample size calculation upon an effect size of 0.65SD (detecting at least a 0.97 unit (log10 scale) higher antibody response in the BTKi suspension group with 90% statistical

For peer review only - http://bmjopen.bmj.com/site/about/guidelines.xhtml

power at 2-sided 5% significance level which requires data from 102 participants. This leads to 120 participants after allowing for 15% missing data. This calculation was performed using the Stata version 15.1 ‘power twomeans’ command.

Statistical analysis Full details will be presented in a separate statistical analysis plan (SAP) which will be drafted and finalised and aimed to be published prior to the final analysis data lock and will receive review and input from the trial steering committee (TSC) and data monitoring committee (DMC). The principal analysis will be performed on the as randomised (“intention to treat”) population, analysing participants with available outcome data in their randomised groups, regardless of adherence. The study will be reported in line with Consolidated Standards of Reporting Trials (CONSORT) guidelines [35].

The primary objective of the statistical analysis will be to identify if a temporary 3 week pausing of BTKi around the time of vaccination against SARS-CoV-2 increases the anti-spike-RBD antibody at 3 weeks post vaccine compared to continuing BTKi treatment without interruption. The anti-spike-RBD antibody levels will be summarised descriptively at baseline and 3, and 12-weeks post COVID-19 booster vaccination follow-up. The differences between the study arms will be estimated using a mixed effects regression model, allowing for repeated measures clustered within participants. The model will be adjusted for the randomisation factor (first line or subsequent line of therapy), and prior infection status obtained from pre-vaccination anti-nucleocapsid antibodies, as fixed effects. A treatment by time interaction will be included. The model is anticipated to use an unstructured covariance matrix, and maximum likelihood estimation. Data will be log-transformed prior to analysis, as appropriate. The adjusted mean difference between the groups will be presented, together with 95% CI and p-values. The regression model will be simplified (e.g. linear regression) if the planned model does not converge.

For peer review only - http://bmjopen.bmj.com/site/about/guidelines.xhtml

Supplemental analyses will explore the time effect between the last COVID-19 vaccination dose and the dose given in IMPROVE. The effect of non-compliance to the randomised intervention will be explored using per-protocol analyses.

The same model as for the primary outcome will be used to analyse the 12 week PVB anti-spike-RBD antibody outcome. Similar supportive analyses as planned for the primary outcome will be performed for 12 week PVB data. 50% and 90% neutralisation by Wuhan and VOC along with, T cell spike response by subtype (spike for Wuhan and VOC, and nucleocapsid and membrane) will be analysed in a similar manner to anti-spike RBD antibody outcome. Other secondary outcomes will be analysed using generalised linear models for binary and continuous data, as appropriate, with model adjustment as described above.

The number of SAEs will be presented by treatment arm. The proportion of participants with at least one SAE will be compared. Details of the events will be presented, together with information on the timing of the events from randomisation.

Missing data will be described with reasons given where available; the number and percentage of individuals in the missing category will be presented by treatment arm. No data will be considered spurious in the analysis since all data will be checked and cleaned before analysis.

Timing of analysis: The final unblinded (to the study's non-statistician investigators) statistical analysis will take place after all follow-up has been completed, and sufficient time has been allowed for data collection and cleaning. No formal interim statistical analyses are planned or have been allowed for in the study design.

The trial statisticians will have access to the final dataset. Once the study has been completed and the main finding have been published, the CI will also have access to the final study dataset.

Patient and public involvement

For peer review only - http://bmjopen.bmj.com/site/about/guidelines.xhtml

People with CLL have been involved since inception of the study. Discussion with a small group (6 people) of people with CLL confirmed the trial question is of considerable relevance to the CLL community and advised on study design. A PPI representative (LD) is a co-applicant on the grant, a member of the trial management group, and a non-independent member of the trial steering committee. A Patient Advisory Group has been convened and will meet regularly throughout the study. The role of this group is to work with the IMPROVE study team to ensure that the perspective of people living with CLL is considered during the design, conduct and reporting of the research. This will be achieved through the group helping to raise awareness of the study, reviewing documents, inputting on any issues requested by the trial team, and advising on dissemination of the study results to the CLL community.

Trial oversight and management

The study is sponsored by the University of Birmingham. Day to day management of the study and all programming and statistical activities are to be undertaken by the UKCRC Registered Trials Unit – Oxford Clinical Trials Research Unit (OCTRU) at the University of Oxford.

For peer review only

For peer review only - http://bmjopen.bmj.com/site/about/guidelines.xhtml

Discussion

The IMPROVE study is designed to investigate in those with Chronic Lymphocytic Leukaemia on BTKi therapy for more than 1 year if a 3 week pause in therapy, compared to continuing as usual with BTKi therapy, improves immune responses to COVID-19 booster vaccination. It will assess the quantitative and qualitative humoral response, to ancestral B.1 virus and the latest variant of concern. In addition, cellular immunity will be assessed through T cell responses to ancestral B.1 and omicron variant peptide pools to give a comprehensive assessment of the adapted immune response. It is generally accepted that both binding and neutralising antibodies are correlates of protection against SARS-CoV-2 symptomatic disease, and the contribution of cellular immunity can be important in protection from severe disease amongst those that are immunocompromised. The study will generate data that will be generalisable for patients taking one of the currently licensed BTKi in the UK and may be applicable to patients taking this therapy for other conditions, where treatment can be safely interrupted. Treatment interruption can be associated with disease flare, and as such, data outcomes of this will be collected over the course of the study in order for the risk/benefit to be assessed to inform future clinical decision making.

As this study focuses on patients with CLL, we have chosen to use the internationally validated EORTC QLQ-CLL17 [32]. This will identify symptom burden, physical condition/fatigue, and worries/fears on health and functioning and allow direct comparison with other published data.

Non-blinding of the participants is a potential limitation to the findings, although concordance with study arm will be assessed through the 3 week CRF. Importantly, laboratory personnel will be blinded to the allocation for objective primary and secondary endpoints and, as such, the results should provide clarity for patients and clinicians around any beneficial impact on immunity of pausing BTKi therapy around the time of vaccination. The published results will help to inform national and international treatment guidance.

Ethics and Dissemination This study has been approved by Leeds East Research Ethics Committee and Health Research Authority (REC Reference:22/YH/0226, IRAS: 319057). It will be

For peer review only

publicised to research, clinical and patient communities and other important stakeholders. This paper has been produced using the SPIRIT reporting guidelines [36]. Once the study is completed, we aim to publish the study results in a peer-reviewed high impact journal and present at national and international meetings to ensure maximum impact and rapid dissemination. The study's PPI partners will advise on the content of all public facing content for dissemination and where else the study's results should be communicated. There will be no restrictions on the publication of study findings. All authors will be required to meet the ICJME authorship requirements. Participant level dataset and statistical code will be made available to bona-fide researchers upon request from OCTRU and the CI, once the IMPROVE study findings have been published in full. The full study protocol may be accessed from the NIHR website.

Details of study monitoring arrangements and a history of changes to the protocol is available in the article supplementary material.

For peer review only

Footnotes:

Twitter: @ProfJACook, @OCTRUctu

Contributors: VSB wrote the first draft of the protocol paper. HMP is the Chief investigator of the study and conceived it. HMP, LD, AA, PM, JAC are grant holders. JAC provided statistical expertise in clinical trial design and NP is conducting the primary statistical analysis. AF led the trials unit delivery of the study. All co-authors reviewed it critically, contributed intellectually, and approved the final version.

Funding: The study was funded by the National Institute for Health Research (NIHR) – Efficacy Mechanism Evaluation programme under the reference NIHR151892 (https://fundingawards.nihr.ac.uk/award/NIHR151892). The views expressed in this document are those of the author(s) and not necessarily those of the NIHR or the Department of Health and Social Care. The study is sponsored by The University of Birmingham, Birmingham, UK (researchgovernance@contacts.bham.ac.uk), and is being managed by the Oxford Clinical Trials Research Unit (OCTRU) at the University of Oxford. The study sponsor and funders do not have any role in study design; collection, management, analysis, and interpretation of data; writing of the report; and the decision to submit the report for publication. The study funder and sponsor do not have authority over any of these activities.

Competing interests: The institutions of all co-authors received funding from the NIHR-MRC-EME programme (NIHR151892) for conducting this research. In addition, HMP reports research grants from Medical Research Council, NIHR and National Core Studies in the past 36 months and advisory fees and personal payments for lectures for AstraZeneca, Janssen and Abbvie pharmaceuticals. AA reports institutional research grants from Astra Zeneca and Oxford Immunotec, personal author royalties from UpTodate and Springer, personal consulting fees from Inflazome and NGM Biopharmaceuticals, personal payments for lectures from Menarini pharmaceuticals and Cadilla pharmaceuticals, in the previous 36 months, and unrelated to the

For peer review only - http://bmjopen.bmj.com/site/about/guidelines.xhtml

current work. AA, JAC and LD report research grants from the NIHR programmes in the last 36 months.

Figure 1. Participant flow in the IMPROVE study

REFERENCES

1. CRUK. *Chronic lymphocytic leukaemia (CLL) incidence statistics* 2015 12/01/2022].
2. Hallek, M., *Chronic lymphocytic leukemia: 2017 update on diagnosis, risk stratification, and treatment.* Am J Hematol, 2017. **92**(9): p. 946-965.
3. National Cancer Institute Surveillance, E., and End Results Programme *Cancer Stat Facts: Leukemia - Chronic Lymphocytic Leukemia (CLL).* [cited 2023 19 June]; Available from: https://seer.cancer.gov/statfacts/html/clyl.html.
4. Sharma, S. and K.R. Rai, *Chronic lymphocytic leukemia (CLL) treatment: So many choices, such great options.* Cancer, 2019. **125**(9): p. 1432-1440.
5. Milne, K., B. Sturrock, and T. Chevassut, *Chronic Lymphocytic Leukaemia in 2020: the Future Has Arrived.* Curr Oncol Rep, 2020. **22**(4): p. 36.
6. Watson, L., P. Wyld, and D. Catovsky, *Disease burden of chronic lymphocytic leukaemia within the European Union.* Eur J Haematol, 2008. **81**(4): p. 253-8.
7. Molica, S., *Sex differences in incidence and outcome of chronic lymphocytic leukemia patients.* Leuk Lymphoma, 2006. **47**(8): p. 1477-80.
8. *HMRN Incidence* 08/05/2023]; Available from: hmrn.org/statistics/incidence.
9. Lee A R Y B, W.S.Y., Chai L Y A, Lee S C, Lee M X, Muthiah M D et al, *Efficacy of covid-19 vaccines in immunocompromised patients: systematic review and meta-analysis.* BMJ, 2022. **376**: p. e068632.
10. Molica, S., D. Levato, and L. Levato, *Infections in chronic lymphocytic leukemia. Analysis of incidence as a function of length of follow-up.* Haematologica, 1993. **78**(6): p. 374-7.
11. Molica, S., *Infections in chronic lymphocytic leukemia: risk factors, and impact on survival, and treatment.* Leuk Lymphoma, 1994. **13**(3-4): p. 203-14.
12. Andersen, M.A., et al., *Incidence and predictors of infection among patients prior to treatment of chronic lymphocytic leukemia: a Danish nationwide cohort study.* Haematologica, 2018. **103**.
13. Paneesha, S., et al., *Covid-19 infection in therapy-naive patients with B-cell chronic lymphocytic leukemia.* Leuk Res, 2020. **93**: p. 106366.
14. Scarfo, L., et al., *COVID-19 severity and mortality in patients with chronic lymphocytic leukemia: a joint study by ERIC, the European Research Initiative on CLL, and CLL Campus.* Leukemia, 2020. **34**(9): p. 2354-2363.
15. Mato, A.R., et al., *Outcomes of COVID-19 in patients with CLL: a multicenter international experience.* Blood, 2020. **136**(10): p. 1134-1143.
16. Noori, M., et al., *A systematic review and meta-analysis of immune response against first and second doses of SARS-CoV-2 vaccines in adult patients with hematological malignancies.* Int Immunopharmacol, 2022. **110**: p. 109046.
17. Jimenez, M., et al., *Kinetics of cellular and humoral immunogenicity and effectiveness of SARS-CoV-2 booster vaccination in hematologic neoplasms.* Am J Hematol, 2023.
18. Mauro, F.R., et al., *Response to the conjugate pneumococcal vaccine (PCV13) in patients with chronic lymphocytic leukemia (CLL).* Leukemia, 2021. **35**(3): p. 737-746.
19. Pleyer, C., et al., *Effect of Bruton tyrosine kinase inhibitor on efficacy of adjuvanted recombinant hepatitis B and zoster vaccines.* Blood, 2021. **137**(2): p. 185-189.
20. van der Velden, A.M., et al., *Influenza virus vaccination and booster in B-cell chronic lymphocytic leukaemia patients.* Eur J Intern Med, 2001. **12**(5): p. 420-424.

For peer review only - http://bmjopen.bmj.com/site/about/guidelines.xhtml

21. Zent, C.S., et al., *Short term results of vaccination with adjuvanted recombinant varicella zoster glycoprotein E during initial BTK inhibitor therapy for CLL or lymphoplasmacytic lymphoma.* Leukemia, 2021. **35**(6): p. 1788-1791.
22. Herishanu, Y., et al., *Six-month antibody persistence after BNT162b2 mRNA COVID-19 vaccination in patients with chronic lymphocytic leukemia.* Blood Adv, 2022. **6**(1): p. 148-151.
23. Moreno, C., et al., *Restoration of the immune function as a complementary strategy to treat Chronic Lymphocytic Leukemia effectively.* J Exp Clin Cancer Res, 2021. **40**(1): p. 321.
24. Abhishek, A., et al., *Effect of a 2-week interruption in methotrexate treatment versus continued treatment on COVID-19 booster vaccine immunity in adults with inflammatory conditions (VROOM study): a randomised, open label, superiority trial.* Lancet Respir Med, 2022. **10**(9): p. 840-850.
25. Hampel, P.J., et al., *Disease Flare During Temporary Interruption of Ibrutinib Therapy in Patients with Chronic Lymphocytic Leukemia.* Oncologist, 2020. **25**(11): p. 974-980.
26. Hou, J.Z., et al., *Real-world ibrutinib dose reductions, holds and discontinuations in chronic lymphocytic leukemia.* Future Oncol, 2021. **17**(35): p. 4959-4969.
27. Parry, H., et al., *Impaired neutralisation of SARS-CoV-2 delta variant in vaccinated patients with B cell chronic lymphocytic leukaemia.* J Hematol Oncol, 2022. **15**(1): p. 3.
28. Greenberger, L.M., et al., *Anti-spike antibody response to SARS-CoV-2 booster vaccination in patients with B cell-derived hematologic malignancies.* Cancer Cell, 2021. **39**(10): p. 1297-1299.
29. Hallek, M., et al., *iwCLL guidelines for diagnosis, indications for treatment, response assessment, and supportive management of CLL.* Blood, 2018. **131**(25): p. 2745-2760.
30. Agency, U.H.S., *COVID-19 vaccination programme - documents relating to the COVID-19 vaccination programme* Crown
31. Ahn, I.E., et al., *Clinically indicated ibrutinib dose interruptions and reductions do not compromise long-term outcomes in CLL.* Blood, 2019. **133**(22): p. 2452-2455.
32. Oerlemans, S., et al., *International validation of the EORTC QLQ-CLL17 questionnaire for assessment of health-related quality of life for patients with chronic lymphocytic leukaemia.* Br J Haematol, 2022. **197**(4): p. 431-441.
33. Duggan, J.O., A.; Andrews, N., *Evaluation of Roche Elecsys anti-SARS-CoV-2 S serology assay for the detection of anti-SARS-CoV-2 S antibodies*, P.H. England, Editor. 2021, Crown.
34. Group, N.S.-C.-S.A.E., *Performance characteristics of five immunoassays for SARS-CoV-2: a head-to-head benchmark comparison.* Lancet Infect Dis, 2020. **20**: p. 1390-400.
35. Schulz, K.F., et al., *CONSORT 2010 statement: updated guidelines for reporting parallel group randomised trials.* BMJ, 2010. **340**: p. c332.
36. Chan, A.W., et al., *SPIRIT 2013 explanation and elaboration: guidance for protocols of clinical trials.* BMJ, 2013. **346**: p. e7586.

For peer review only

Table 1: IMPROVE study research assessments at different time-points

**Assessments**	**Pre-Vaccine (baseline)**	**Vaccine date known**	**1 week before Vaccination**	**At Vaccination**	**1 week after Vaccination**	**3 weeks after Vaccination**	**12 weeks after Vaccination**
Demographics	*						
Height and weight	*						
Current medication	*						
Comorbidities	*						
Past SARS-CoV-2 vaccines	*						
Serum immunoglobins	*						
Disease activity	*					*	*
Quality of life	*					*	*
Randomisation to include a check that no monoclonal antibody has been received since baseline visit		*					
Reminders of allocation to continue or withhold BTKi			*	*	*		
Adherence to intervention						*	
Check on any monoclonal antibody (prophylaxis or treatment for COVID-19)		*				*	*
Safety						*	*
Details of vaccination						*	
Provision of aide memoire card to participant	*						
Thank you note sent after visit	*					*	*
**Samples**	**Pre-Vaccine (baseline)**	**Vaccine date known**	**1 week before Vaccination**	**At Vaccination**	**1 week after Vaccination**	**3 weeks after Vaccination**	**12 weeks after Vaccination**
Blood sample taken for anti-spike RBD antibody	*					*	*
Blood sample taken for T-cell ELISPOT	*					*	
Blood sample taken for blood film	*					*	*

For peer review only - http://bmjopen.bmj.com/site/about/guidelines.xhtml

Blood sample taken for neutralisation assay	*					*	
Blood sample taken for LDH measurement	*					*	*
Blood sample taken for full blood count measurement	*					*	*
Blood sample taken to be banked for mechanistic work as per section 11				§	§	* (no extra blood volume required from that already being taken)	* (no extra blood volume required from that already being taken)

§ To be taken from 20 participants only

For peer review only - http://bmjopen.bmj.com/site/about/guidelines.xhtml

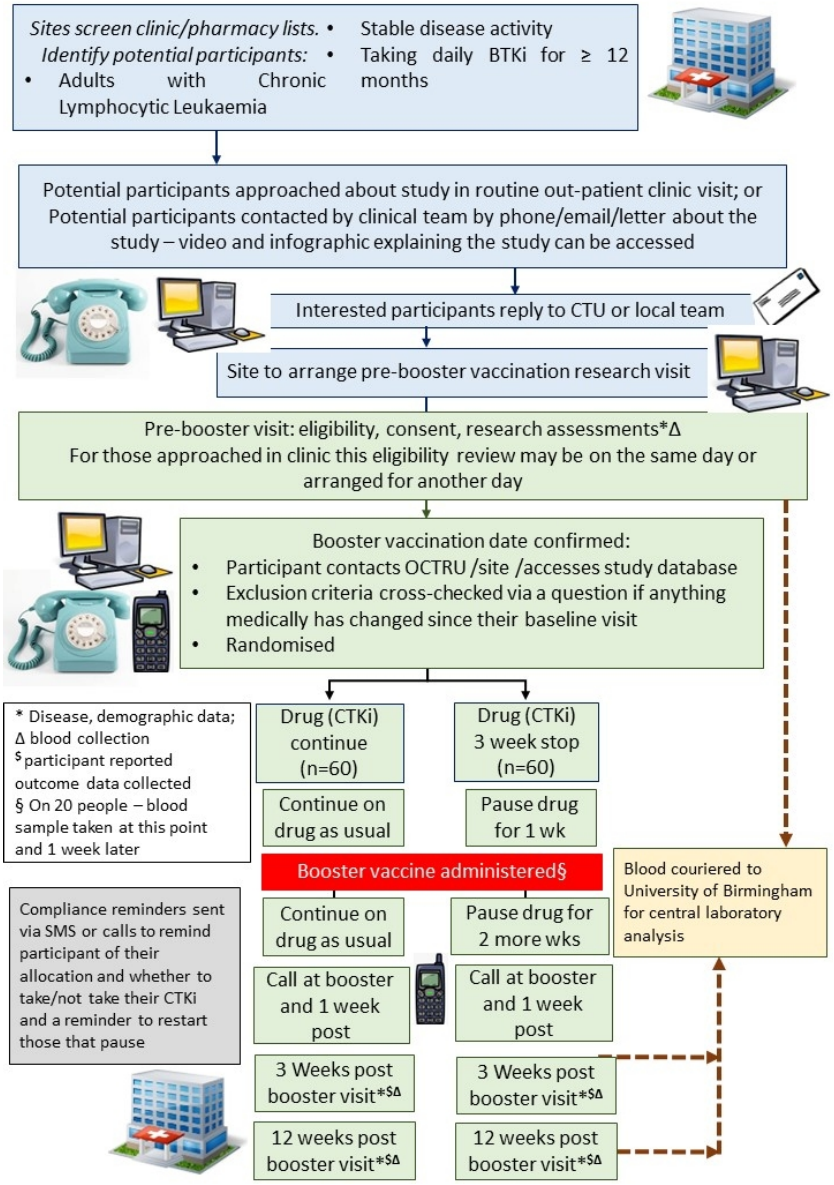

Participant flow in the IMPROVE study

190x275mm (96 x 96 DPI)

Supplementary material:

1. Monitoring

Data Monitoring Committee (DMC) A DMC comprising of a group of three independent experts including at least one statistician and one clinician, external to the study will be constituted. It will assess the progress, conduct and critical outcomes of the study and will meet regularly throughout the study at time-points agreed by the Chair of the Committee and the CI. The DMC will review the safety data generated, including all serious adverse events, and make recommendations as to whether the protocol should be amended to protect patient safety. It will report to the chair of the TSC via the trial statistician. The DMC is independent of the sponsor and will maintain a list of conflicts of interest.

Auditing: The Oxford Clinical Trials and Research Unit (OCTRU) Quality Assurance team will undertake periodic audits on behalf of the sponsor.

Study management

The Chief Investigator has overall responsibility for the study and shall oversee all study management. The data custodian will be the Chief Investigator.

Trial Steering Committee (TSC) The role of the independent TSC is to provide the overall supervision of the study. They will monitor the study's progress and conduct, and will advise on scientific credibility. The committee will consider and act, as appropriate, upon the recommendations of the DMC and ultimately carries the responsibility for deciding whether the study needs to be stopped on grounds of safety or efficacy. The committee includes independent members and members of the research team.

Trial Management Group (TMG) consists of those individuals responsible for the operational management of the study such as the chief investigator and co-investigators, Trials group

For peer review only - http://bmjopen.bmj.com/site/about/guidelines.xhtml

operational lead, the trial manager and the trial statistician. Other specialities/ individuals will be invited as required for specific items/issues.

The TMG will meet usually at least once a month throughout the lifetime of the study and will:

- Supervise the conduct and progress of the study, and adherence to the study protocol;
- Assess the safety as compiled by the study team and assessed by the DMC;
- Evaluate the quality of the study data.

2. History of changes

Protocol version no.	Protocol date	Summary of key changes from previous version
1.0	18Aug2022	1st version of the protocol.
2.0	15Sep2022	Typographical errors updated and removal of remote consent
3.0	20Sep2022	Typographical errors updated and clarification of labelling on research blood samples and update of funder logo and insertion of ISRCTN allocated
4.0	08Nov2022	Reduction of text in one of the text messages due to limit of text system for 460 characters.<br>Minor changes to text messages.<br>Correction of who reply slips are returned to.<br>Addition of window for collection of extra optional samples<br>Change to timepoint of collection of vaccination history.<br>Typographical corrections
5.0	24Mar2023	Addition of aide memoire card.<br>Update to outcome measure for secondary endpoints to include influenza vaccination given during Autumn 2023.<br>Typographical corrections.<br>Addition of funder statement to front page and study duration to synopsis (requested by funder)

For peer review only - http://bmjopen.bmj.com/site/about/guidelines.xhtml

Reporting checklist for protocol of a clinical trial.

Based on the SPIRIT guidelines.

Instructions to authors

Complete this checklist by entering the page numbers from your manuscript where readers will find each of the items listed below.

Your article may not currently address all the items on the checklist. Please modify your text to include the missing information. If you are certain that an item does not apply, please write "n/a" and provide a short explanation.

Upload your completed checklist as an extra file when you submit to a journal.

In your methods section, say that you used the SPIRITreporting guidelines, and cite them as:

Chan A-W, Tetzlaff JM, Gøtzsche PC, Altman DG, Mann H, Berlin J, Dickersin K, Hróbjartsson A, Schulz KF, Parulekar WR, Krleža-Jerić K, Laupacis A, Moher D. SPIRIT 2013 Explanation and Elaboration: Guidance for protocols of clinical trials. BMJ. 2013;346:e7586

		Reporting Item	Page Number
**Administrative information**			
Title	#1	Descriptive title identifying the study design, population, interventions, and, if applicable, trial acronym	1
Trial registration	#2a	Trial identifier and registry name. If not yet registered,	3

For peer review only

		name of intended registry	
Trial registration: data set	#2b	All items from the World Health Organization Trial Registration Data Set	On ISRCTN record
Protocol version	#3	Date and version identifier	3
Funding	#4	Sources and types of financial, material, and other support	19
Roles and responsibilities: contributorship	#5a	Names, affiliations, and roles of protocol contributors	19
Roles and responsibilities: sponsor contact information	#5b	Name and contact information for the trial sponsor	19
Roles and responsibilities: sponsor and funder	#5c	Role of study sponsor and funders, if any, in study design; collection, management, analysis, and interpretation of data; writing of the report; and the decision to submit the report for publication, including whether they will have ultimate authority over any of these activities	19
Roles and responsibilities: committees	#5d	Composition, roles, and responsibilities of the coordinating centre, steering committee, endpoint adjudication committee, data management team, and	26

For peer review only

		other individuals or groups overseeing the trial, if applicable (see Item 21a for data monitoring committee)	
**Introduction**			
Background and rationale	#6a	Description of research question and justification for undertaking the trial, including summary of relevant studies (published and unpublished) examining benefits and harms for each intervention	4
Background and rationale: choice of comparators	#6b	Explanation for choice of comparators	4
Objectives	#7	Specific objectives or hypotheses	5
Trial design	#8	Description of trial design including type of trial (eg, parallel group, crossover, factorial, single group), allocation ratio, and framework (eg, superiority, equivalence, non-inferiority, exploratory)	6
**Methods: Participants, interventions, and outcomes**			
Study setting	#9	Description of study settings (eg, community clinic, academic hospital) and list of countries where data will be collected. Reference to where list of study sites can be obtained	6

For peer review only

Eligibility criteria	#10	Inclusion and exclusion criteria for participants. If applicable, eligibility criteria for study centres and individuals who will perform the interventions (eg, surgeons, psychotherapists)	7
Interventions: description	#11a	Interventions for each group with sufficient detail to allow replication, including how and when they will be administered	9
Interventions: modifications	#11b	Criteria for discontinuing or modifying allocated interventions for a given trial participant (eg, drug dose change in response to harms, participant request, or improving / worsening disease)	9
Interventions: adherance	#11c	Strategies to improve adherence to intervention protocols, and any procedures for monitoring adherence (eg, drug tablet return; laboratory tests)	9 and 12
Interventions: concomitant care	#11d	Relevant concomitant care and interventions that are permitted or prohibited during the trial	12
Outcomes	#12	Primary, secondary, and other outcomes, including the specific measurement variable (eg, systolic blood pressure), analysis metric (eg, change from baseline, final value, time to event), method of aggregation (eg, median, proportion), and time point for each outcome. Explanation of the clinical relevance of chosen efficacy and harm outcomes is strongly recommended	10

For peer review only

Participant timeline	#13	Time schedule of enrolment, interventions (including any run-ins and washouts), assessments, and visits for participants. A schematic diagram is highly recommended (see Figure)	11 and 25
Sample size	#14	Estimated number of participants needed to achieve study objectives and how it was determined, including clinical and statistical assumptions supporting any sample size calculations	14
Recruitment	#15	Strategies for achieving adequate participant enrolment to reach target sample size	6
**Methods: Assignment of interventions (for controlled trials)**			
Allocation: sequence generation	#16a	Method of generating the allocation sequence (eg, computer-generated random numbers), and list of any factors for stratification. To reduce predictability of a random sequence, details of any planned restriction (eg, blocking) should be provided in a separate document that is unavailable to those who enrol participants or assign interventions	8
Allocation concealment mechanism	#16b	Mechanism of implementing the allocation sequence (eg, central telephone; sequentially numbered, opaque, sealed envelopes), describing any steps to conceal the	8

For peer review only

		sequence until interventions are assigned	
Allocation: implementation	#16c	Who will generate the allocation sequence, who will enrol participants, and who will assign participants to interventions	8
Blinding (masking)	#17a	Who will be blinded after assignment to interventions (eg, trial participants, care providers, outcome assessors, data analysts), and how	9
Blinding (masking): emergency unblinding	#17b	If blinded, circumstances under which unblinding is permissible, and procedure for revealing a participant's allocated intervention during the trial	N/A
**Methods: Data collection, management, and analysis**			
Data collection plan	#18a	Plans for assessment and collection of outcome, baseline, and other trial data, including any related processes to promote data quality (eg, duplicate measurements, training of assessors) and a description of study instruments (eg, questionnaires, laboratory tests) along with their reliability and validity, if known. Reference to where data collection forms can be found, if not in the protocol	11
Data collection plan: retention	#18b	Plans to promote participant retention and complete follow-up, including list of any outcome data to be	9 and 12

For peer review only

		collected for participants who discontinue or deviate from intervention protocols	
Data management	#19	Plans for data entry, coding, security, and storage, including any related processes to promote data quality (eg, double data entry; range checks for data values). Reference to where details of data management procedures can be found, if not in the protocol	12
Statistics: outcomes	#20a	Statistical methods for analysing primary and secondary outcomes. Reference to where other details of the statistical analysis plan can be found, if not in the protocol	15
Statistics: additional analyses	#20b	Methods for any additional analyses (eg, subgroup and adjusted analyses)	16
Statistics: analysis population and missing data	#20c	Definition of analysis population relating to protocol non-adherence (eg, as randomised analysis), and any statistical methods to handle missing data (eg, multiple imputation)	15
**Methods: Monitoring**			
Data monitoring: formal committee	#21a	Composition of data monitoring committee (DMC); summary of its role and reporting structure; statement of whether it is independent from the sponsor and competing interests; and reference to where further details about its charter can be found, if not in the protocol. Alternatively, an explanation of why a DMC is	26

For peer review only

		not needed	
Data monitoring: interim analysis	#21b	Description of any interim analyses and stopping guidelines, including who will have access to these interim results and make the final decision to terminate the trial	17
Harms	#22	Plans for collecting, assessing, reporting, and managing solicited and spontaneously reported adverse events and other unintended effects of trial interventions or trial conduct	12 and 16
Auditing	#23	Frequency and procedures for auditing trial conduct, if any, and whether the process will be independent from investigators and the sponsor	N/A
**Ethics and dissemination**			
Research ethics approval	#24	Plans for seeking research ethics committee / institutional review board (REC / IRB) approval	2
Protocol amendments	#25	Plans for communicating important protocol modifications (eg, changes to eligibility criteria, outcomes, analyses) to relevant parties (eg, investigators, REC / IRBs, trial participants, trial registries, journals, regulators)	27
Consent or assent	#26a	Who will obtain informed consent or assent from potential trial participants or authorised surrogates, and	11

For peer review only

		how (see Item 32)	
Consent or assent: ancillary studies	#26b	Additional consent provisions for collection and use of participant data and biological specimens in ancillary studies, if applicable	11
Confidentiality	#27	How personal information about potential and enrolled participants will be collected, shared, and maintained in order to protect confidentiality before, during, and after the trial	14
Declaration of interests	#28	Financial and other competing interests for principal investigators for the overall trial and each study site	20 (for central trial team)
Data access	#29	Statement of who will have access to the final trial dataset, and disclosure of contractual agreements that limit such access for investigators	12
Ancillary and post trial care	#30	Provisions, if any, for ancillary and post-trial care, and for compensation to those who suffer harm from trial participation	N/A
Dissemination policy: trial results	#31a	Plans for investigators and sponsor to communicate trial results to participants, healthcare professionals, the public, and other relevant groups (eg, via publication, reporting in results databases, or other data sharing arrangements), including any publication restrictions	19
Dissemination policy:	#31b	Authorship eligibility guidelines and any intended use of	19

For peer review only

authorship		professional writers	
Dissemination policy: reproducible research	#31c	Plans, if any, for granting public access to the full protocol, participant-level dataset, and statistical code	19

Appendices

Informed consent materials	#32	Model consent form and other related documentation given to participants and authorised surrogates	Available from the trial team
Biological specimens	#33	Plans for collection, laboratory evaluation, and storage of biological specimens for genetic or molecular analysis in the current trial and for future use in ancillary studies, if applicable	13 and 14

None The SPIRIT Explanation and Elaboration paper is distributed under the terms of the Creative Commons Attribution License CC-BY-NC. This checklist can be completed online using https://www.goodreports.org/, a tool made by the EQUATOR Network in collaboration with Penelope.ai

For peer review only
