# Supplementary material for: Protocol for a multicentre randomised controlled trial examining the effects of temporarily pausing Bruton tyrosine kinase inhibitor therapy to coincide with SARS-CoV-2 vaccination and its impact on immune responses in patients with chronic lymphocytic leukaemia
Source: BMJ Open. 2023 Sep 28;13(9):e077946. doi: 10.1136/bmjopen-2023-077946 (PMC10546125; doi:10.1136/bmjopen-2023-077946)
Supplement: Reviewer comments [file bmjopen-2023-077946.reviewer_comments.pdf]

PEER REVIEW HISTORY

BMJ Open publishes all reviews undertaken for accepted manuscripts. Reviewers are asked to complete a checklist review form (**http://bmjopen.bmj.com/site/about/resources/checklist.pdf**) and are provided with free text boxes to elaborate on their assessment. These free text comments are reproduced below.

ARTICLE DETAILS

**TITLE (PROVISIONAL)**	Protocol for a multi-centre randomised controlled trial examining the effects of temporarily pausing Bruton Tyrosine Kinase inhibitor therapy to coincide with SARS-CoV-2 vaccination and its impact on immune responses in patients with Chronic Lymphocytic Leukaemia
**AUTHORS**	Barber, Vicki; Peckham, Nicholas; Duley, Lelia; Francis, Anne; Abhishek, Abhishek; Moss, Paul; Cook, Jonathan; Parry, Helen

This article was not externally reviewed at BMJ Open. Protocols that have been independently assessed prior to submission to BMJ Open are usually fast-tracked to publication on the grounds that further substantial changes will not be possible. This independent assessment will usually be external, independent review for both a substantial grant award from a non-commercial or government funder and ethics approval.
